# Multi-Locus Genome-Wide Association Study of Four Yield-Related Traits in Chinese Wheat Landraces

**DOI:** 10.3389/fpls.2021.665122

**Published:** 2021-08-16

**Authors:** Yu Lin, Kunyu Zhou, Haiyan Hu, Xiaojun Jiang, Shifan Yu, Qing Wang, Caixia Li, Jian Ma, Guangdeng Chen, Zisong Yang, Yaxi Liu

**Affiliations:** ^1^State Key Laboratory of Crop Gene Exploration and Utilization in Southwest China, Chengdu, China; ^2^Triticeae Research Institute, Sichuan Agricultural University, Chengdu, China; ^3^School of Life Science and Technology, Henan Institute of Science and Technology, Xinxiang, China; ^4^College of Resources, Sichuan Agricultural University, Chengdu, China; ^5^College of Resources and Environment, Aba Teachers University, Wenchuan, China

**Keywords:** validation, wheat landraces, candidate genes, quantitative trait loci, association study, yield-related traits

## Abstract

Wheat (*Triticum aestivum* L.) is one of the most important crops in the world. Here, four yield-related traits, namely, spike length, spikelets number, tillers number, and thousand-kernel weight, were evaluated in 272 Chinese wheat landraces in multiple environments. Five multi-locus genome-wide association studies (FASTmrEMMA, ISIS EN-BLASSO, mrMLM, pKWmEB, and pLARmEB) were performed using 172,711 single-nucleotide polymorphisms (SNPs) to identify yield-related quantitative trait loci (QTL). A total of 27 robust QTL were identified by more than three models. Nine of these QTL were consistent with those in previous studies. The remaining 18 QTL may be novel. We identified a major QTL, *QTkw.sicau-4B*, with up to 18.78% of phenotypic variation explained. The developed kompetitive allele-specific polymerase chain reaction marker for *QTkw.sicau-4B* was validated in two recombinant inbred line populations with an average phenotypic difference of 16.07%. After combined homologous function annotation and expression analysis, *TraesCS4B01G272300* was the most likely candidate gene for *QTkw.sicau-4B*. Our findings provide new insights into the genetic basis of yield-related traits and offer valuable QTL to breed wheat cultivars *via* marker-assisted selection.

## Introduction

Wheat (*Triticum aestivum* L.) is one of the most important crops in the world. A 100% increase in crop production by 2050 will be needed to keep pace with projected human population growth (Ray et al., 2013). Thus, it is imperative to increase crop yield. Wheat yield consists of three main components, including spike number per plant, grain number per spike, and thousand-kernel weight (TKW). Spike number per plant is determined by tillers number (TN) per plant. Spikelets number (SN) per spike and spike length (SL) significantly affect grain number per spike. Understanding the genetic basis of these yield-related traits can contribute to improving wheat yield.

Chinese wheat landraces have been widely used for breeding cultivated varieties of wheat (Dai et al., 2009). Wheat landraces show high genetic diversity and extensive phenotypic variation, such as early maturity, high numbers of kernel per spikelet, and good adaption to local environmental conditions (He and Huang, 2001; Hao et al., 2008). Genetic analyses of Chinese wheat landraces have revealed the basis of agronomic traits, such as yield-related traits (spikelets number per spike, tillers number, and thousand-kernel weight), plant morphological traits (flag leaf length, flag leaf width, and plant height; Liu et al., 2017; Ma et al., 2020), stress resistance (pre-harvest sprouting, drought-related traits, and phosphorus-deficiency tolerance; Zhou et al., 2017; Lin et al., 2019, 2020a), and disease resistance (powdery mildew and stripe rust; Xiao et al., 2013; Long et al., 2019). Analysis of gene diversity and polymorphism information content revealed the high diversity of Chinese wheat landraces (Liu et al., 2017; Long et al., 2019). Thus, genetic analysis of yield traits using Chinese wheat landraces can provide important insights into wheat breeding.

With the development of next-generation sequencing (NGS), genome-wide association study (GWAS) has become an effective way of detecting complex quantitative characteristics and is also widely applied in *Arabidopsis* (Atwell et al., 2010; Bac-Molenaar et al., 2016), rice (Huang et al., 2010; Zhu et al., 2016), maize (Lu et al., 2010, 2012; Yang et al., 2014), *Aegilops tauschii* (Liu et al., 2015a,b; Qin et al., 2015, 2016), and wheat (Liu et al., 2014, 2017; Maccaferri et al., 2015; Sukumaran et al., 2015; Lin et al., 2017). Moreover, the previous studies have discovered genes *via* GWAS directly. In rice, Yano identified a gene comprehensively controlling rice architecture using GWAS (Yano et al., 2019). Kim reported a novel resistance gene, *Xa43(t)*, for bacterial blight (Kim and Reinke, 2019). In particular, GWAS has gradually been applied to wheat landraces. Using GWAS, a total of 149 significant markers for 21 agronomic traits were detected in 723 wheat landraces (Liu et al., 2017). A total of 51 loci significantly associated with adult-plant resistance to stripe rust were discovered in wheat landrace through GWAS (Long et al., 2019). Recently, a major locus of coleoptile length on chromosome 6B was revealed by GWAS in 707 Chinese wheat landraces (Ma et al., 2020).

Because of the mass of data involved in the process of GWAS, several multi-locus models have been designed to increase efficiency (Wang et al., 2016). Compared with the single-locus model, the multi-locus models can help improve the detection power of GWAS (Xu et al., 2018). Therefore, multi-locus models have recently been popularized in plant GWAS, such as in the photosynthetic response to low phosphorus stress in soybean (Lü et al., 2018), fatty acid content in rapeseed (Guan et al., 2019), forage quality-related traits in sorghum (Li et al., 2018), salt tolerance of direct seeding in rice (Cui et al., 2018), callus regenerative traits, starch pasting properties, and stalk lodging resistance-related traits in maize (Ma et al., 2018; Xu et al., 2018; Zhang et al., 2018), agronomic traits in barley (Hu et al., 2018), and free amino acid levels in wheat (Peng et al., 2018). All these studies have successfully discovered novel quantitative trait loci (QTL).

In the present study, a total of 272 Chinese wheat landraces were evaluated in multiple environments to improve our understanding of the genetic basis of four yield-related traits. Five multi-locus GWAS models were performed to identify robust QTL using 172,711 SNPs. Major QTL were validated in two recombinant inbred line (RIL) populations. Furthermore, we presumed candidate genes for the major QTL. This study provides new QTL of yield-related traits that may help wheat breading in the near future.

## Materials and Methods

### Plant Material, Phenotype Evaluation, and Data Analysis

A total of 272 wheat landraces, obtained from 10 major wheat-growing zones in China, were utilized in this study (Supplementary Table S1). All landraces were planted in six environments: Ya’an (103°370 E, 22°420 N) in 2012 (E1), Wenjiang (103°410 E, 30°360 N) in 2013, 2014, and 2015 (E2, E3, and E4), and Chongzhou (103°390 E, 30°330 N) in 2014 and 2015 (E5 and E6). Each row of material was 30 cm apart and 1.5 m long, and contained 15 seeds. Field management referred to criteria commonly practiced in wheat production. Four yield-related traits were evaluated in at least four environments: SL – the average length of the main spikes from five plants; SN – the average total number of spikelets from five main spikes of the plant; TN – the average number of tillers in five plants; TKW – the average weight of five samples of 1,000 kernels randomly selected from a given genotype. Two bi-parental populations [Huimai × Datianquxiaomai (HD) and Huimai × Heshangmai (HH)] were used to validate the results. The parents of bi-parental populations were selected from 272 wheat landraces. The TKW of these two RIL populations was evaluated in Chongzhou in 2019, and Huimai resulted significantly more productive than Datianquxiaomai and Heshangmai (higher TKW).

To eliminate environmental effects, the best linear unbiased prediction (BLUP) value for each trait was calculated across environments and used for statistical analysis. The BLUP was calculated using the methodology of Piepho et al. (2008) as previously described (Liu et al., 2017). The broad-sense heritability (*H*^2^) value was calculated using SAS v8.1 (SAS Institute Inc., Cary, NC, United States) and is defined as:

(1) $$H^{2}=\mathrm{Vg}/\left(\mathrm{Vg}+\mathrm{Vge}+\mathrm{Ve}\right),$$

where V_g_, V_ge_, and V_e_ are the estimates of genetic variance, the genotype × environment interaction, and environmental variance, respectively (Smith et al., 1998). Correlation analyses were performed using SPSS 20 (IBM SPSS Statistics; IBM Corp., Armonk, NY, United States).

### Genotyping

Genomic DNA was extracted from a single plant for each of the accessions using the cetyltrimethylammonium bromide method (Murray and Thompson, 1980). DNA samples with an A260/280 ratio of 1.8–2.0 and a concentration of >80 ng μl^−1^ were used for genotyping. The 272 accessions had been already genotyped by wheat 660 K array, including 630,517 probes (Axiom^®^ Wheat660 SNP array; Affymetrix, Santa Clara, CA, United States) by Zhou et al. (2017). The software, bowtie2, was employed in this study to obtain physical locations of 172,711 SNPs based on IWGSC RefSeq v1.0.^1^

### GWAS for Yield Traits

The population structure was performed by Structure2.3.4 based on the Bayesian clustering algorithm (Pritchard et al., 2000). Ten runs of STRUCTURE were performed with a K between 1 and 10 using the admixture model with 100,000 replicates for burn-in and 100,000 replicates for MCMC. The R package “mrMLM” in R Project for Statistical Computing was used to examine the association between markers and yield-related traits. Marker-trait associations were performed using five multi-locus models, including FASTmrEMMA (Wen et al., 2017), ISIS EM-BLASSO (Tamba et al., 2017), mrMLM (Wang et al., 2016), pKWmEB (Ren et al., 2018), and pLARmEB (Zhang et al., 2017). All five models were adjusted by both population structure and family relationship. A logarithm of odds (LOD) value ≥3 was used as the screening criterion (Guan et al., 2019). According to previous studies, the linkage disequilibrium decay of wheat ranges from 3.5 to 23 Mb (Kidane et al., 2019; Li et al., 2019; Luján Basile et al., 2019). Significant SNPs were therefore selected with a physical distance ≤10 Mb and referred to as a conservative QTL.

### Validation of QTL Using Two RIL Populations

To further validate the significant QTL identified for TKW, the peak SNP with this locus was converted into a kompetitive allele-specific polymerase chain reaction (KASP) marker based on the probe sequence. KASP primers were designed and analyzed as described in previous studies (Lin et al., 2020b, 2021) and produced by Sangon Biotech (Shanghai, China; Supplementary Table S2). The KASP marker detected different alleles at this locus in the two bi-parental populations. Eighty-two lines were selected for each of the alleles from both populations. These lines were used to evaluate differences in TKW between the two allele groups using a Student’s t-test in SPSS 20 (IBM SPSS Statistics; IBM Corp., Armonk, NY, United States).

### Candidate Gene Prediction

Based on IWGSC RefSeq v1.0, predicted genes within ~10 Mb of the physical location of the QTL were selected. We undertook two different methods to predict the possible existence of candidate genes. The first method was expression analysis. Based on data from WheatExp,^2^ genes expressed highly at stages Z71 and Z75 were the most important, due to the key stages in kernel development (Zadoks et al., 1974). Fragments per kilobase of exon model (FPKM) represented gene expression level. As in Lin et al. (2020b), genes expressing less than two per million mapped reads at any stage were removed. FPKM is fragments read per thousand bases per million mappings and represented gene expression level.

The second method to predict the possible existence of candidate genes was annotation. All genes were also used to perform homologous annotations in rice and Arabidopsis using the KEGG Orthology Based Annotation System 3.0 (KOBAS 3.0; Xie et al., 2011).^3^ Functional annotations were performed *via* UniProt^4^ and EnsemblPlants.^5^

## Results

### Phenotypic Variation in Chinese Wheat Landraces

The four traits among the 272 wheat landraces varied considerably (Table 1). Based on the BLUP values, the SL ranged from 6.33 to 14.63 (cm). SN ranged from 19.43 to 27.36 (count). TN ranged from 8.11 to 18.10 (count), and TKW ranged from 17.90 to 40.47 (g). The heritability of these four traits ranged from 0.64 to 0.93 (Table 1). TN and TKW showed medium heritability, whereas SL and SN showed high heritability. Correlation analysis showed that all correlations were significant (*p* < 0.05), except the correlation between SL and SN (Table 2), indicating that the changes in these two traits are independent.

**Table 1:** Phenotype variation and heritability of spike length (SL), spikelets number per spike (SN), tillers number (TN), and thousand-kernel weight (TKW) based on BLUP values for the 272 accessions.

Variables	Full trait name	Unit	Tested environment	Range	Mean	SD	CV (%)	Heritability
SL	Spike length	cm	6	6.33–14.63	10.59	1.57	14.83	0.93
SN	Spikelets number per spike	count	6	19.43–27.36	23.28	1.62	6.96	0.92
TN	Tillers number	count	4	8.11–18.1	12.03	1.72	14.3	0.64
TKW	Thousand-kernel weight	g	5	17.9–40.47	29.63	4.12	13.9	0.79

*BLUP, best linear unbiased prediction; SD, standard deviation; CV, coefficients of variation*.

**Table 2:** Correlation of spike length (SL), spikelets number per spike (SN), tillers number (TN), and TKW based on BLUP values.

	SL	SN	TN	TKW
SL	1			
SN	0.07	1		
TN	−0.16^**^	0.36^**^	1	
TKW	0.14^*^	−0.40^**^	−0.53^**^	1

Level of significance: ^*^and ^**^significant at p < 0.05 and p < 0.01, respectively.

### Five Multi-Locus Models of Yield-Related Traits

**Figure 1:**
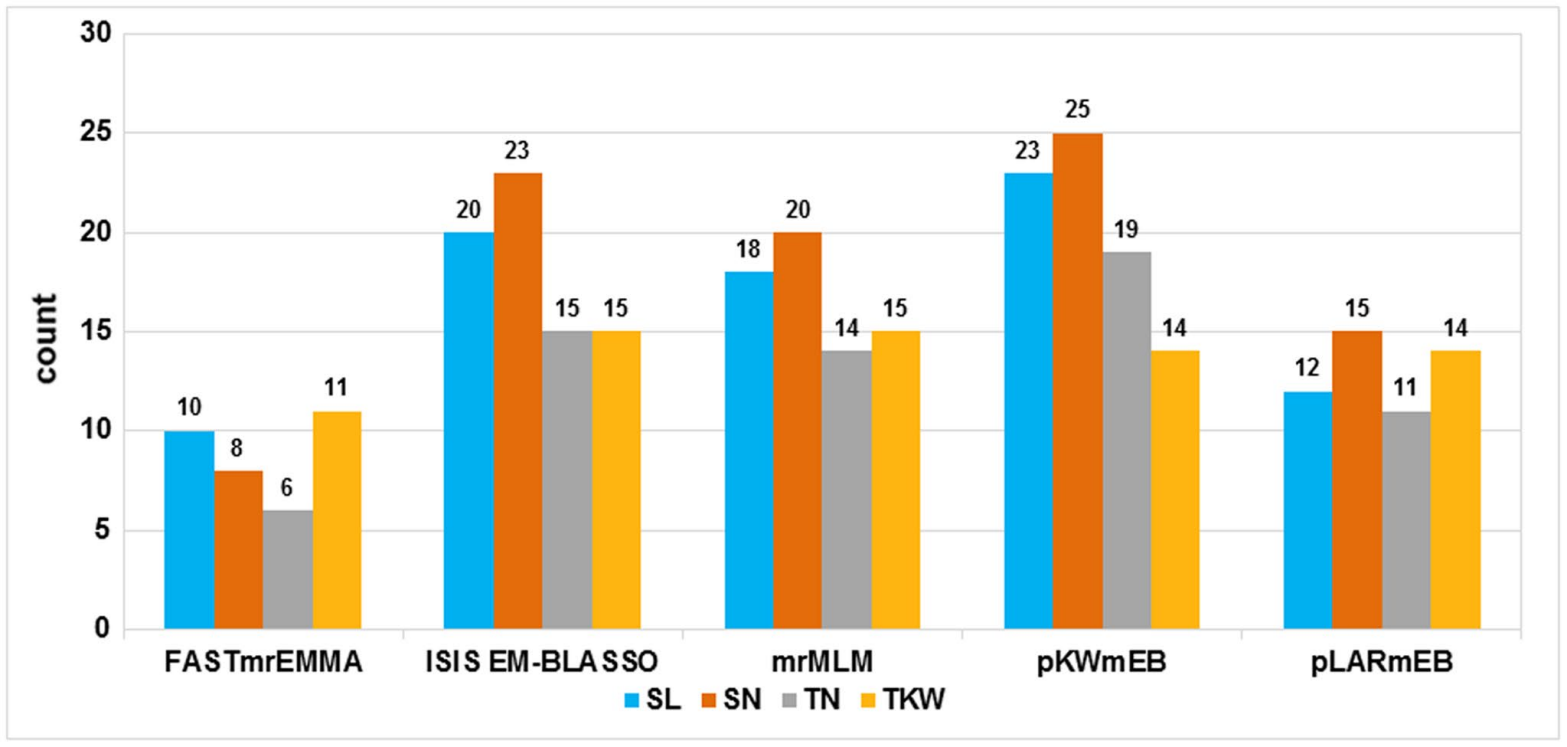
Comparison of the number of detected quantitative trait loci (QTL) from the five methods. The five methods are mrMLM, FASTmrEMMA, ISIS EM-BLASSO, pLARmEB, and pKWmEB. The traits included spike length (SL), spikelets number per spike (SN), thousand-kernel weight, and tillers number (TN). Total denotes the total QTL number for each trait.

A total of 172,711 polymorphic SNPs were obtained [after filtering for missing values ≤10% and minor allele frequency (MAF) ≥0.05] from the Wheat 660 K SNP arrays. This subset was used to perform GWAS. Based on LOD values ≥3, a total of 308 significant SNP markers were identified using the five multi-locus models. Detailed significant SNP markers information and the number of significant SNP markers detected by different models are shown in Supplementary Table S3 and Figure 1, respectively. In FASTmrEMMA, the least number of significant SNP markers was detected, with only 35. A total of 10, 8, 6, and 11 significant SNP markers were detected for SL, SN, TN, and TKW, respectively, with phenotypic variation explained (PVE) up to 6.55%. In the model of ISIS EM-BLASSO, 73 significant SNP markers were revealed. A total of 20, 23, 15, and 15 were detected for SL, SN, TN, and TKW, respectively, with PVE up to 9.27%. The mrMLM model could detect 67 significant markers. A total of 18, 20, 14, and 15 were detected for SL, SN, TN, and TKW, respectively, with PVE up to 13.09%. In the model of pKWmEB, the most number of significant SNP markers was detected (81), and a total of 23, 25, 19, and 14 were detected for SL, SN, TN, and TKW, respectively, with PVE up to 18.78%. The pLARmEB model detected 52 significant SNP markers, and 12, 15, 11, and 14 were detected for SL, SN, TN, and TKW, respectively, with PVE up to 11.09%. Significant markers existed in the above three models, and no more than 10 Mb was considered as a robust QTL.

### Robust QTL Selected by Five Multi-Locus Models

Twenty-seven robust QTL were identified by more than three different multi-locus models and were considered as robust QTL (Table 3).

**Table 3:** Details of robust QTL associated with yield-related traits by multi-locus GWAS.

Variables	QTL	Marker	Chromosome	Marker position (Mb)	Method	LOD	PVE (%)
SL	*QSl.sicau-1A*	AX-110620420	1A	566.11	ISIS EM-BLASSO	7.57	3.01
		AX-110620420	1A	566.11	mrMLM	7.54	4.38
		AX-110375230	1A	568.36	mrMLM	5.63	3.14
SL	*QSl.sicau-1D*	AX-109320713	1D	24.06	FASTmrEMMA	3.45	2.34
		AX-109320713	1D	24.06	ISIS EM-BLASSO	6.86	3.29
		AX-109320713	1D	24.06	pLARmEB	4.65	1.36
SL	*QSl.sicau-2A*	AX-108948074	2A	735.26	ISIS EM-BLASSO	10.11	5.89
		AX-108948074	2A	735.26	pKWmEB	11.56	7.43
		AX-108948074	2A	735.26	pLARmEB	5.23	1.95
		AX-110088953	2A	742.14	ISIS EM-BLASSO	4.74	4.15
		AX-110088953	2A	742.14	mrMLM	12.51	11.44
SL	*QSl.sicau-2B*	AX-111519386	2B	29.19	mrMLM	12.03	7.77
		AX-111519386	2B	29.19	pKWmEB	3.47	2.24
		AX-94545725	2B	29.71	ISIS EM-BLASSO	8.45	3.97
SL	*QSl.sicau-3A*	AX-109461933	3A	650.86	ISIS EM-BLASSO	14.68	4.88
		AX-109461933	3A	650.86	mrMLM	7.03	2.72
		AX-109461933	3A	650.86	pKWmEB	5.66	2.8
		AX-110469735	3A	651.84	pLARmEB	7.36	3.11
SL	*QSl.sicau-4A*	AX-111098528	4A	94.83	mrMLM	3.5	2.75
		AX-109827217	4A	96.05	ISIS EM-BLASSO	5.64	2.19
		AX-109827217	4A	96.05	pKWmEB	6.11	4.03
SL	*QSl.sicau-7B*	AX-109306414	7B	742.41	FASTmrEMMA	5.19	2.74
		AX-109306414	7B	742.41	ISIS EM-BLASSO	4.1	1.56
		AX-109306414	7B	742.41	mrMLM	5.34	3.11
		AX-109306414	7B	742.41	pKWmEB	4.04	3.05
SN	*QSn.sicau-1B*	AX-109305103	1B	630.28	ISIS EM-BLASSO	8.79	2.97
		AX-109305103	1B	630.28	mrMLM	7.21	2.25
		AX-109305103	1B	630.28	pKWmEB	9.5	3.04
SN	*QSn.sicau-2A.1*	AX-108924276	2A	31.70	mrMLM	4.3	3.6
		AX-108924276	2A	31.70	pKWmEB	9.41	4.33
		AX-108831532	2A	32.54	pLARmEB	3.7	1.45
SN	*QSn.sicau-2A.2*	AX-111594388	2A	524.09	mrMLM	8.84	7.02
		AX-111594388	2A	524.09	pLARmEB	4.45	5.03
		AX-111076227	2A	524.34	ISIS EM-BLASSO	7.14	4.76
SN	*QSn.sicau-2A.3*	AX-111123457	2A	741.83	ISIS EM-BLASSO	5.34	3.09
		AX-111123457	2A	741.83	pLARmEB	4.62	4.59
		AX-111450513	2A	741.98	pKWmEB	8.84	5.27
SN	*QSn.sicau-2B.1*	AX-109905931	2B	529.05	ISIS EM-BLASSO	5.3	2.32
		AX-109905931	2B	529.05	pKWmEB	6.59	3.45
		AX-111054119	2B	529.29	mrMLM	3.65	1.11
SN	*QSn.sicau-2B.2*	AX-110604055	2B	650.16	ISIS EM-BLASSO	3.51	1.66
		AX-110604055	2B	650.16	pLARmEB	5.33	2.85
		AX-109340301	2B	653.01	pKWmEB	5.14	2.4
SN	*QSn.sicau-3B*	AX-108940748	3B	644.91	ISIS EM-BLASSO	10.56	5.17
		AX-108940748	3B	644.91	mrMLM	5.6	2.96
		AX-108940748	3B	644.91	pKWmEB	6.95	2.9
SN	*QSn.sicau-5A*	AX-108842302	5A	678.62	pKWmEB	4.31	1.39
		AX-109538487	5A	678.94	ISIS EM-BLASSO	4.55	1.64
		AX-110560000	5A	680.92	mrMLM	6.26	4.49
		AX-110560000	5A	680.92	pKWmEB	4.36	2.83
		AX-109409751	5A	682.90	FASTmrEMMA	3.07	3.43
		AX-109816265	5A	682.92	mrMLM	5.63	2.83
SN	*QSn.sicau-7A*	AX-110931532	7A	671.48	ISIS EM-BLASSO	7.39	5.13
		AX-110931532	7A	671.48	pKWmEB	15.85	7.61
		AX-111600553	7A	671.48	ISIS EM-BLASSO	4.07	2.83
		AX-111600553	7A	671.48	pLARmEB	4.81	5.44
TN	*QTn.sicau-1B*	AX-109582231	1B	21.47	FASTmrEMMA	3.28	1.81
		AX-109582231	1B	21.47	ISIS EM-BLASSO	3.01	1.4
		AX-109582231	1B	21.47	mrMLM	4.47	4.38
		AX-109582231	1B	21.47	pKWmEB	5.12	2.93
		AX-109582231	1B	21.47	pLARmEB	4.6	2.12
TN	*QTn.sicau-2B*	AX-110093452	2B	10.99	ISIS EM-BLASSO	4.27	3.04
		AX-110093452	2B	10.99	pLARmEB	6.1	3.87
		AX-110407616	2B	10.99	pKWmEB	7.76	3.88
TN	*QTn.sicau-5B*	AX-109427123	5B	605.53	ISIS EM-BLASSO	5.3	2.12
		AX-109427123	5B	605.53	mrMLM	4.43	3.56
		AX-109427123	5B	605.53	pKWmEB	3.14	1.74
TN	*QTn.sicau-6D*	AX-110969084	6D	469.60	FASTmrEMMA	3.77	3.7
		AX-110989608	6D	472.57	ISIS EM-BLASSO	4.26	2.96
		AX-110989608	6D	472.57	pKWmEB	8.42	6.73
		AX-111919829	6D	472.69	mrMLM	8.2	9.58
		AX-109296056	6D	472.74	pLARmEB	6.04	3.53
TKW	*QTkw.sicau-2B*	AX-111113548	2B	50.73	ISIS EM-BLASSO	9.88	6.9
		AX-111113548	2B	50.73	pKWmEB	5.39	5.97
		AX-111213423	2B	50.77	FASTmrEMMA	3.37	1.91
TKW	*QTkw.sicau-3A*	AX-95003297	3A	686.12	FASTmrEMMA	4.16	2.13
		AX-95003297	3A	686.12	ISIS EM-BLASSO	5.35	2.35
		AX-95003297	3A	686.12	mrMLM	5.39	3.02
		AX-95003297	3A	686.12	pKWmEB	9.22	4.65
		AX-95003297	3A	686.12	pLARmEB	8.72	3.69
TKW	*QTkw.sicau-4B*	AX-108886949	4B	553.54	ISIS EM-BLASSO	8.59	9.27
		AX-108886949	4B	553.54	pKWmEB	12.04	18.78
		AX-108886949	4B	553.54	pLARmEB	10.2	11.09
TKW	*QTkw.sicau-6B*	AX-111086205	6B	201.58	FASTmrEMMA	7.51	3.54
		AX-111086205	6B	201.58	ISIS EM-BLASSO	3.81	1.39
		AX-111086205	6B	201.58	mrMLM	4.21	2.72
		AX-111086205	6B	201.58	pKWmEB	8.8	5.12
TKW	*QTkw.sicau-7A.1*	AX-109033661	7A	89.32	ISIS EM-BLASSO	5.19	3.18
		AX-109033661	7A	89.32	mrMLM	9.4	6.41
		AX-109033661	7A	89.32	pKWmEB	4.58	4.72
TKW	*QTkw.sicau-7A.2*	AX-110378610	7A	696.41	ISIS EM-BLASSO	5.79	5.1
		AX-110378610	7A	696.41	pKWmEB	4.72	4.71
		AX-110378610	7A	696.41	pLARmEB	8.35	5.63
TKW	*QTkw.sicau-7B.1*	AX-110982569	7B	517.43	FASTmrEMMA	5.3	2.75
		AX-110982569	7B	517.43	mrMLM	6.25	3.03
		AX-110982569	7B	517.43	pLARmEB	7.16	2.99

*SL, spike length; SN, spikelets number per spike; TN, tillers number; TKW, thousand-kernel weight; LOD, logarithm of odds; PVE, phenotypic variation explained*.

Seven QTL associated with SL were identified on chromosomes 1A, 1D, 2A, 2B, 3A, 4A, and 7B, with the PVE up to 11.44%, and the LOD values ranging from 3.45 to 14.68. *QSl.sicau-3A,* located at 650.86–651.84 Mb, was identified in four models, with LOD values up to 14.68. Nine QTL associated with SN were identified on chromosomes 1B, 2A, 2B, 3B, 5A, and 7A, with PVE up to 7.61%, and LOD values up to 15.85. *QSn.sicau-7A*, located at 671.48 Mb, was detected by four models. The highest LOD (15.85) and PVE (7.61%) values on this QTL were detected by the pKWmEB model. Four QTL for TN were identified on chromosomes 1B, 2B, 5B, and 6D, with a PVE up to 9.58%, and the LOD values up to 8.42. *QTn.sicau-6D*, located at 469.60–472.74 Mb, had the highest PVE value which was 9.58%. Seven QTL associated with TKW were identified on chromosomes 2B, 3A, 4B, 6B, 7A, and 7B, with PVE values up 18.78%, and the LOD values up to 12.04. *QTkw.sicau-4B*, identified by the pKWmEB model, explained the highest PVE (18.78%). Interestingly, among all robust QTL detected in the present study, *QTkw.sicua-4B* resulted that with the highest PVE value. It can be regarded as a major QTL, contributing to breed more productive wheat cultivars, so it was validated in the first instance.

### Validation of Genetic Effect and Candidate Genes of *QTkw.sicau-4B*

**Figure 2:**
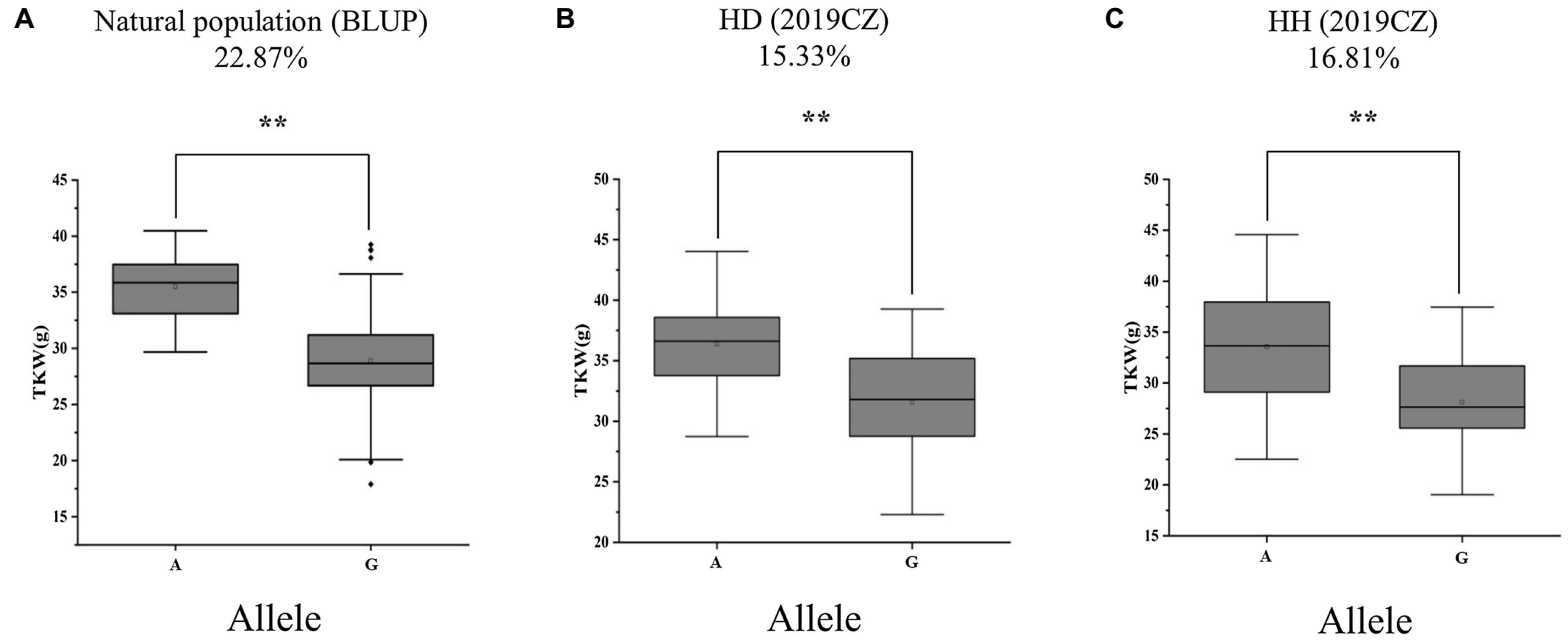
Alleles effects of *QTkw.sicau-4B* in **(A)** natural **(B)** HD, and **(C)** HH populations. HD: Huimai × Datianquxiaomai; HH: Huimai × Heshangmai; BLUP, best linear unbiased prediction; and 2019CZ: in Chongzhou in 2019. ^**^ significant at *p* < 0.01.

To validate the genetic effect of the peak SNP for *QTkw.sicau-4B*, a KASP marker (KASP-AX-108886949) was developed and the differences between TKW of landraces carrying alternatively A/G allele were calculated, both in natural population and in two RIL populations. Among the 272 landraces tested, the average TKW of landraces carrying genotype allele A was heavier than those carrying allele G (*p* < 0.01; Table 4; Figure 2A). The difference was 22.87%. In the HD population, genotypes were divided into two groups: 42 with allele A and 40 with allele G. The TKW of genotypes with allele A ranged from 28.75 to 44.04 g, whereas those with allele G ranged from 22.28 to 39.27 g. The average TKW with allele A was heavier than that with allele G (*p* < 0.01; Table 4; Figure 2B). In the RIL population HH, there were 39 with allele A and 43 with allele G. The TKW of genotypes with allele A ranged from 22.52 to 44.56 (g), whereas those with allele G ranged from 19.03 to 37.47 g. The average TKW with allele A was heavier than with allele G (*p* < 0.01; Table 4; Figure 2C). The difference in TKW between genotypes ranged from 15.33 to 16.81%, with an average value of 16.07% among the two RIL populations. Thus, the developed KASP marker may be useful for breeding cultivars carrying a high-TKW allele.

**Table 4:** Allele effect of *QTkw.sicau-4B* in two RILs population.

	HD (2019CZ)	HH (2019CZ)	BLUP of natural population
*p*-value	**	**	**
Average TKW of allele A (g)	36.39	33.56	35.49
Average TKW of allele G (g)	31.55	28.73	28.89
Range of TKW in population (g)	22.28–44.04	19.03–44.56	17.90–40.47
Difference (%)	15.33	16.81	22.87

HD, Huimai × Datianquxiaomai; HH, Huimai × Heshangmai; BLUP, best linear unbiased prediction; and ^**^ significant at p < 0.01.

**Figure 3:**
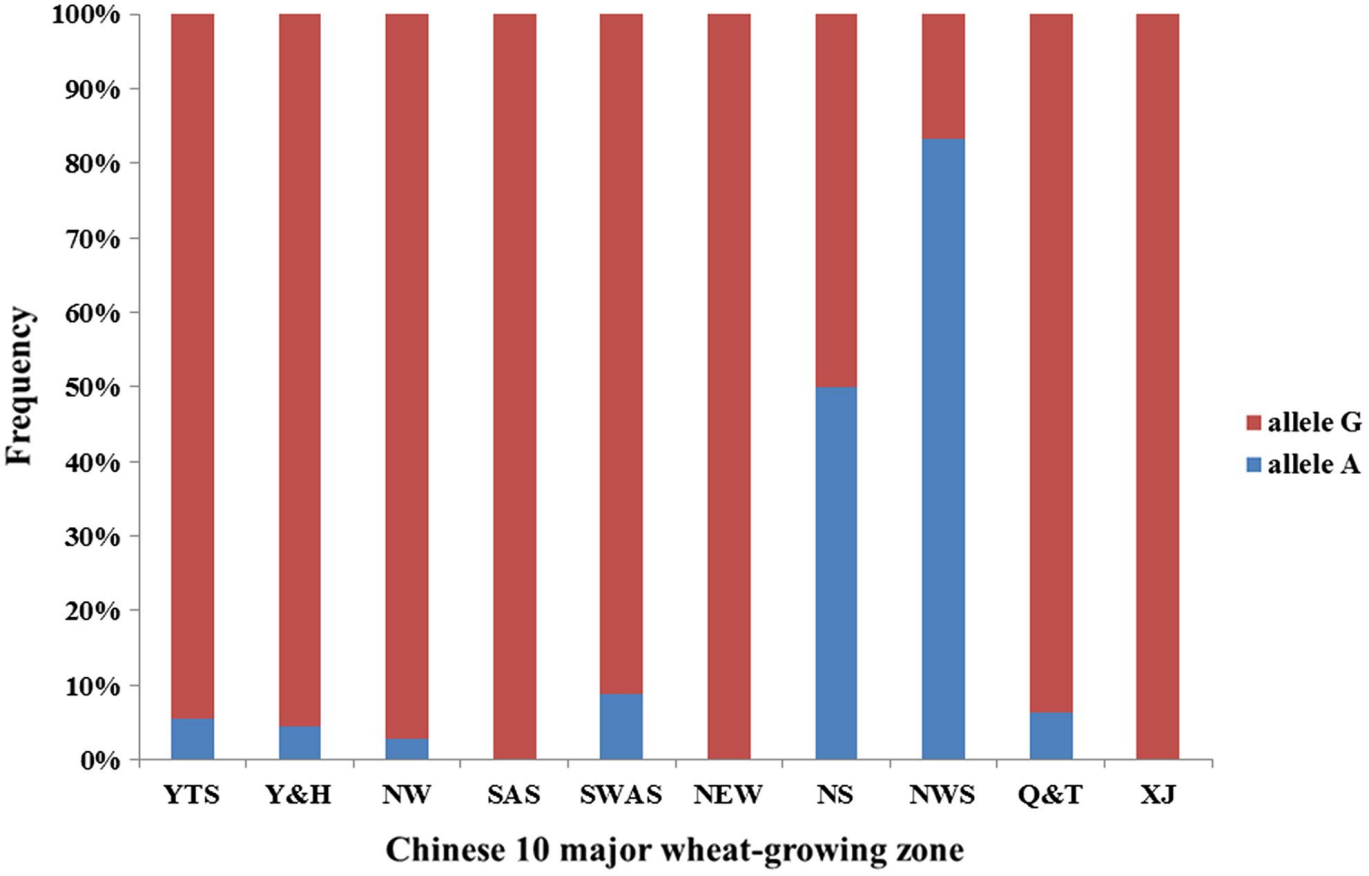
Allele frequency of *QTkw.sicau-4B* in 10 major wheat-growing zones. Blue means allele A proportion in Chinese wheat landrace and red means allele G proportion in Chinese wheat landrace. NW (Northern Winter Wheat Zone), Y&H (Yellow and Huai River Valleys Facultative Wheat Zone), YTS (Middle and Low Yangtze Valleys Autumn-Sown Spring Wheat Zone), SAS (Southern Autumn-Sown Spring Wheat Zone), SWAS (Southwestern Autumn-Sown Spring Wheat Zone), NES (Northeastern Spring Wheat Zone), NS (Northern Spring Wheat Zone), NWS (Northwestern Spring Wheat Zone), Q&T (Qinghai-Tibetan Plateau Spring–Winter Wheat Zone), and XJ (Xinjiang Winter–Spring Wheat Zone).

We observed superior allele A frequency in 10 major wheat-growing zones (Figure 3). The frequencies show that the superior allele A showed an 8% frequency in Chinese wheat landraces, and more than half the materials from the Northern Spring Wheat Zone and the Northwestern Spring Wheat Zone carried allele A. The Northern Winter Wheat Zone, Yellow and Huai River Valleys Facultative Wheat Zone, Middle and Low Yangtze Valleys Autumn-Sown Spring Wheat Zone, Southwestern Autumn-Sown Spring Wheat Zone, and Qinghai-Tibetan Plateau Spring–Winter also had some materials with allele A. Materials from the Southern Autumn-Sown Spring Wheat Zone, Northeastern Spring Wheat Zone, and Xinjiang Winter–Spring Wheat Zone did not carry allele A.

A total of 46 high-confidence genes which were identified in IWGSC RefSeq v1.0 flanked *QTkw.sicau-4B* by no more 5 Mb. The search for candidate genes was undertaken following two pipelines. The expression analysis of the candidates was investigated in the two most interesting stages for kernel development: Z71 and Z75. Nine genes were expressed at both stages, namely, *TraesCS4B01G272300, TraesCS4B01G272500, TraesCS4B01G273400,* Traes*CS4B01G273500, TraesCS4B01G274200, TraesCS4B01G274500, TraesCS4B01G275400, TraesCS4B01G275500,* and *TraesCS4B01G276300*. Based on these sequences, we found homologous genes using KOBAS 3.0 (see footnote 3). Three genes were identified as TKW-regulating candidate genes (Table 5). *TraesCS4B01G272300, TraesCS4B01G275400,* and *TraesCS4B01G276200* were homologous to *OsMCA1* (Liu et al., 2015c), *H2B* (Ma et al., 2019a), and *EDR1* (Prasad et al., 2009) in rice, respectively.

**Table 5:** List of three candidate genes flanking *QTkw.sicau-4B*.

Candidate gene	Homologous gene in rice	Name	Reference
*TraesCS4B01G272300*	osa:4331673	*OsMCA1*	Liu et al., 2015c
*TraesCS4B01G275400*	osa:4345904	*H2B*	Ma et al., 2019a
*TraesCS4B01G276200*	osa:4331696	*OsCDR1*	Prasad et al., 2009

## Discussion

Yield has always been a focus of global wheat research. With the development of NGS technology, GWAS has been applied extensively to detect complex traits (Liu et al., 2014; Maccaferri et al., 2015; Sukumaran et al., 2015). Previous studies found that the accuracy and efficiency of models, including generalized linear, mixed linear, and multi-locus models, have constantly improved (Zhang et al., 2005; Price et al., 2006; Li et al., 2018). In the present study, we observed not consistent results between different multi-locus models. FASTmrEMMA was more conservative, and pKWmEB was the most effective model of the five models (Supplementary Table S3; Figure 1); this finding is in agreement with the previous studies (Li et al., 2018; Lü et al., 2018). QTL detected by multiple, multi-locus models are considered as robust QTL useful for more precise studies. Among the 308 significant SNP markers, 27 reliable and robust QTL were identified in four yield-related traits in this study.

Using BLAST against the IWGSC RefSeq v1.0, we tried to determine the physical location of QTL flanking markers. Among these 27 robust QTL, nine loci (*QSl.sicau-1A, QSl.sicau-2A, QSl.sicau-2B, QSn.sicua-2A.1, QSn.sicau-2A.3, QSn.sicua-5A, QSn.sicau-7A, QTn.sicua-6D,* and *QTkw.sicau-3A*) were overlapped with, or located close to, previously reported QTL.

For the trait of SL, three QTL identified in present study were reported previously (*QSl.sicau-1A, QSl.sicau-2A,* and *QSl.sicau-2B*). QTL for SL located at 572.23 to 572.35 Mb, between markers Xmwg632 and Xbarc213 (Yu et al., 2014), were close to *QSl.sicau-1A* (566.11–568.36 Mb). *QSl.sicau-2A* (735.26–742.14 Mb) resulted overlapping with *QEl.fcu-2A*, reported by Faris et al. (2014) and flanked by Xwmc181 (728.6 1 Mb) and fcp651 (738.43 Mb) markers. Moreover, *QSl.sicau-2B* (29.19–29.71 Mb) overlapped with the previous reported QTL: *Qsl2B.2* flanking by Xbarc200 at 26.59 Mb (Xu et al., 2014) and flanking by *excalibur_c40567_1893* at 28.37 Mb (Zhai et al., 2016).

For SN, four robust QTL identified in the present study were reported previously. *QSns.sau-2A.1* (Ma et al., 2019b) exists between markers AX-111610554 and AX-110495160, located at 35.78 to 36.00 Mb, and is close to our *QSn.sicua-2A.1* (31.70–32.45 Mb). *QSn.sicau-2A.3* for SN located on chromosome 2A at 741.83–741.98 Mb resulted close to *QCmp.fcu-2A* identified between Xwmc181 and Xfcp651 at 728.60–738.44 Mb (Faris et al., 2014). Wang et al. (2011) discovered a robust QTL controlling spikelets number per spike in three environments; the QTL detected with marker Xgwm126 (671.4 Mb) and Xgwm291 (698.2 Mb), and overlapped with *QSn.sicua-5A* (678.62–682.92 Mb) in the present study. *QSn.sicau-7A,* located on chromosome 7A at 671.48 Mb, is close to *TaAPO1*, located at 674.08 Mb, and confirmed to play an important role in regulating SN (Kuzay et al., 2019; Ma et al., 2019b; Muqaddasi et al., 2019).

For TN, Bilgrami et al. (2020) reported a QTL *MQTL6D-4* in physical position 456.46–469.25 Mb with a mean R^2^ of 14.08%, which is close to *QTn.sicau-6D* (469.60–472.74 Mb) in the present study.

For TKW, using a doubled haploid population Zhang et al. (2014) identified a QTL for TKW on chromosome 3A (*Qtkw3A-1*) at 625.79–690.79 Mb, which is close to *QTkw.sicau-3A*, and mapped at 686.12 Mb in this study. In general, nine QTL were identified in the same position as those in previous studies; the remaining 18 are potentially novel QTL.

Among robust and novel QTL, we focused on QTkw.sicau-4B that showed the highest PVE value. To validate this major QTL for TKW, we developed a KASP marker according to the sequence of peak marker, AX-108886949 (Supplementary Table S2). According to high PVE value and low frequency, we believe this QTL could help wheat breeding.

Three genes identified by functional annotation showed relationship with TKW. *TraesCS4B01G275400* is orthologous to *H2B* (Histone H2B monoubiquitination), which regulates abscisic acid signaling, and is a target of *UBP26,* which acts as a transcriptional repressor involved in kernel development (Ma et al., 2019a). *TraesCS4B01G276200* is orthologous to *OsCDR1*. Weights between *OsCDR1* transgenic lines and wild-type plants differed (Prasad et al., 2009). *TraesCS4B01G272300* is orthologous to rice OsMCA1, which mutants showed obviously affected TKW (Liu et al., 2015c). *TraesCS4B01G272300* gene, highly expressed in the grain development stage (Supplementary Figure S1), appears to be the most promising candidate gene for *QTkw.sicau-4B*. In our further study, expressions of this candidate gene will be validated by qRT-PCR in different grain development stage. Transgenic tests will also be applied to validate its function.

## Data Availability Statement

The original contributions presented in the study are included in the article/Supplementary Material, further inquiries can be directed to the corresponding author.

## Author Contributions

YL and KYZ drafted and revised the manuscript and contributed to data analysis. HYH, JXJ, SFY, and QW performed the phenotypic evaluation and helped with data analysis. CXL, JM, GDC, and ZSY helped to draft the manuscript. YXL designed and coordinated the study and revised the manuscript. All authors have read and approved the final manuscript for publication.

## Conflict of Interest

The authors declare that the research was conducted in the absence of any commercial or financial relationships that could be construed as a potential conflict of interest.

## Publisher’s Note

All claims expressed in this article are solely those of the authors and do not necessarily represent those of their affiliated organizations, or those of the publisher, the editors and the reviewers. Any product that may be evaluated in this article, or claim that may be made by its manufacturer, is not guaranteed or endorsed by the publisher.

## Footnotes

^1^ http://www.wheatgenome.org/

^2^ https://wheat.pw.usda.gov/WheatExp/

^3^ http://kobas.cbi.pku.edu.cn/kobas3

^4^ https://www.uniprot.org

^5^ http://plants.ensembl.org/index.html
