# Supplementary material for: Multi-Locus Genome-Wide Association Study of Four Yield-Related Traits in Chinese Wheat Landraces
Source: Front Plant Sci. 2021 Aug 16;12:665122. doi: 10.3389/fpls.2021.665122 (PMC8415402; doi:10.3389/fpls.2021.665122)
Supplement: Supplementary file 1 [file Table_1.pdf]

Table S1. Details information of 272 natural population materials for genome-wide association analysis.

Accession-ID	Accession-name	Province	Chinese wheat plant zone[a]	Group[b]
AS661005	baixiaomai	beijing	NW	Group1
AS661006	baimangmai	beijing	NW	Group1
AS661009	tumai	beijing	NW	Group1
AS661012	henanbai	beijing	NW	Group1
AS661016	daqingmang	tianjin	NW	Group1
AS661017	hongmangbai	tianjin	NW	Group1
AS661018	baihulutou	tianjin	NW	Group1
AS661019	xiaobaimang	hebei	Y&H	Group1
AS661021	youzimai	hebei	Y&H	Group1
AS661024	baipibai	hebei	NW	Group1
AS661027	xiaomaimang	hebei	Y&H	Group1
AS661030	gongxianmai	hebei	Y&H	Group1
AS661035	baike	hebei	NW	Group1
AS661037	dabaimai	hebei	Y&H	Group1
AS661038	dabailing	hebei	Y&H	Group1
AS661039	zijingbai	hebei	NW	Group1
AS661041	xiaobaipi	hebei	Y&H	Group1
AS661042	liulengmai	hebei	Y&H	Group1
AS661046	erguangtou	hebei	NW	Group1
AS661054	daqitou	hebei	NW	Group1
AS661056	tutoubai	hebei	NW	Group1
AS661065	dabaimang	hebei	NW	Group1
AS661066	chunqiumaizi	hebei	NW	Group1
AS661071	hongmangbai	hebei	Y&H	Group1
AS661073	baimangmai	hebei	Y&H	Group1
AS661074	guchengxiaomai	hebei	Y&H	Group1
AS661075	gaochan1hao	hebei	Y&H	Group1
AS661076	honghulubaimai	hebei	Y&H	Group1
AS661078	dahongmang	hebei	Y&H	Group1
AS661079	baikebai	hebei	Y&H	Group1
AS661080	baihulutou	hebei	Y&H	Group1
AS661097	xiaomangmai	shanxi	NW	Group1
AS661104	baimanghong	shanxi	NW	Group1
AS661110	baixianmang	shanxi	NW	Group1
AS661112	youmangshangeda	shanxi	NW	Group1
AS661116	baishanmai	shanxi	NW	Group1
AS661119	jinbaoyu	shangdong	Y&H	Group1
AS661120	banmangmai	shandong	Y&H	Group1
AS661123	bo’erqing	shandong	Y&H	Group1
AS661126	hongmangxiaomai	shangdong	Y&H	Group1
AS661129	xiaogongxian	shandong	Y&H	Group1
AS661132	baishamai	shandong	Y&H	Group1
AS661134	xiaobiansui	shandong	Y&H	Group1
AS661137	baitutou	shangdong	Y&H	Group1
AS661138	banjiemangdabai	shandong	Y&H	Group1
AS661139	mazhatoumai	shangdong	Y&H	Group1
AS661140	hongmanghongmai	shangdong	Y&H	Group1
AS661145	baikumai	shangdong	Y&H	Group1
AS661150	hongheshang	shandong	Y&H	Group1
AS661162	iaohongmangliangm	shandong	Y&H	Group1
AS661163	piaoxiao	shangdong	Y&H	Group1

Table S1. Details information of 272 natural population materials for genome-wide association analysis.

Accession-ID	Accession-name	Province	Chinese wheat plant zone[a]	Group[b]
AS661164	baitumai	shangdong	Y&H	Group1
AS661166	hongduomai	shangdong	Y&H	Group1
AS661174	yulincao	shangdong	Y&H	Group1
AS661179	baimangyouzi	henan	Y&H	Group1
AS661182	baigedatou	henan	Y&H	Group1
AS661187	xiaozibai	henan	Y&H	Group1
AS661205	wuhuatou	henan	Y&H	Group1
AS661208	laochushanbao	henan	Y&H	Group1
AS661221	zihuatou	henan	Y&H	Group1
AS661243	shihuiyaohuomai	shaanxi	NW	Group1
AS661251	nv'erhong	shaanxi	NW	Group1
AS661256	gouweibabaimai	shaanxi	Y&H	Group1
AS661259	laobaitiaozi	shaanxi	NW	Group1
AS661281	xiaohongmang	heilongjiang	NES	Group1
AS661284	xuemai	jilin	NES	Group1
AS661287	xinjinyoumang	liaoning	NW	Group1
AS661289	xiaozhihongmai	liaoning	NW	Group1
AS661335	baiqimai	gansu	NW	Group1
AS661337	xiaoqingmang	gansu	NW	Group1
AS661357	kuchebaidongmai	xinjiang	XJ	Group1
AS661445	baisuihong	jiangsu	YTS	Group1
AS661453	xiaoyuhua	jiangsu	YTS	Group1
AS661695	xiaobaipi	inner mongolia	NS	Group1
AS661725	yejihong	shangdong	Y&H	Group1
AS661824	baijiantiao	shaanxi	NW	Group1
AS661980	dingrichangmang	tibet	Q&T	Group1
AS661103	zhuganqing	shanxi	NW	Group2
AS661149	baikangyangmai	shangdong	Y&H	Group2
AS661191	kumai	henan	Y&H	Group2
AS661194	pushanbamai	henan	Y&H	Group2
AS661203	baitiaoyu	henan	Y&H	Group2
AS661207	tutoumai	henan	Y&H	Group2
AS661216	youmangcao	henan	Y&H	Group2
AS661217	dalihong	henan	Y&H	Group2
AS661222	benmai	henan	Y&H	Group2
AS661224	qumangmai	henan	Y&H	Group2
AS661226	xiaobaimai	henan	YTS	Group2
AS661231	baiheshangtou	henan	Y&H	Group2
AS661233	yerenmao	henan	Y&H	Group2
AS661234	xiaozihong	henan	Y&H	Group2
AS661236	youmangcao	henan	YTS	Group2
AS661237	huomai	henan	YTS	Group2
AS661239	baihuomai	henan	Y&H	Group2
AS661380	jianzituan	jiangsu	YTS	Group2
AS661389	huanghuazhu	jiangsu	YTS	Group2
AS661392	yejihong	jiangsu	YTS	Group2
AS661396	huoshaotian	jiangsu	YTS	Group2
AS661398	guangdezao	jiangsu	YTS	Group2
AS661412	nuopangtou	jiangsu	YTS	Group2
AS661414	daoshuibai	jiangsu	YTS	Group2
AS661416	baizhaoyu	jiangsu	YTS	Group2

Table S1. Details information of 272 natural population materials for genome-wide association analysis.

**Accession-ID**	**Accession-name**	**Province**	**Chinese wheat plant zone**[a]	**Group**[b]
AS661421	qingmangzi	jiangsu	YTS	Group2
AS661423	huaqixiaomai	jiangsu	YTS	Group2
AS661425	manghuazi	jiangsu	YTS	Group2
AS661428	baimangzi	jiangsu	YTS	Group2
AS661430	dahongpi	jiangsu	YTS	Group2
AS661435	zhuantoubaike	jiangsu	Y&H	Group2
AS661436	hongmai	jiangsu	Y&H	Group2
AS661440	xiaolihong	jiangsu	Y&H	Group2
AS661452	shunshuihong	jiangsu	YTS	Group2
AS661454	putaomai	shanghai	YTS	Group2
AS661455	baijiaomai	shanghai	YTS	Group2
AS661460	huxumai	anhui	YTS	Group2
AS661461	wugongmai	anhui	YTS	Group2
AS661462	jiangxizao	anhui	YTS	Group2
AS661463	wugongbian	anhui	YTS	Group2
AS661473	dujiaqiaoxiaomai	zhejiang	YTS	Group2
AS661477	baixu	zhejiang	YTS	Group2
AS661479	baikeguangtou	zhejiang	YTS	Group2
AS661483	hongkewugong	zhejiang	YTS	Group2
AS661498	tiezhuantou	zhejiang	YTS	Group2
AS661502	xianjumai	zhejiang	YTS	Group2
AS661507	qingtianmai	zhejiang	YTS	Group2
AS661510	hongtoumai	zhejiang	YTS	Group2
AS661511	xiyangmai	zhejiang	YTS	Group2
AS661521	wugongmai	zhejiang	YTS	Group2
AS661527	tiegengqing	zhejiang	YTS	Group2
AS661528	zaoxiaomai	zhejiang	YTS	Group2
AS661542	qiyanghongke	hunan	YTS	Group2
AS661547	heshangmai	hunan	YTS	Group2
AS661549	baikeyoumang	fujian	SAS	Group2
AS661554	youmai	guangdong	SAS	Group2
AS661555	datianquxiaomai	guangdong	SAS	Group2
AS661558	qingyuanxiaomai	guangdong	SAS	Group2
AS661560	huoshaomai	guangdong	SAS	Group2
AS661562	baikexiaomai	guangdong	SAS	Group2
AS661563	zaoxiaomai	guangdong	SAS	Group2
AS661566	zhangmuxiaomai	guangxi	SAS	Group2
AS661569	guangtoumai	guangxi	SAS	Group2
AS661602	hongxumai	sichuan	SWAS	Group2
AS661626	baikejiang	sichuan	SWAS	Group2
AS661655	baimaier	sichuan	SWAS	Group2
AS661671	guangtoumai	guizhou	SWAS	Group2
AS661675	hongmangmai	guizhou	SWAS	Group2
AS661676	liulengmai	guizhou	SWAS	Group2
AS661684	yangmaizi	yunnan	SWAS	Group2
AS661703	baiyuhua	jiangsu	YTS	Group2
AS661705	nanjinghuang	jiangsu	YTS	Group2
AS661706	zhenjiang3-96	jiangsu	YTS	Group2
AS661715	heshangmai	fujian	SAS	Group2
AS661717	guangtoumai	jiangxi	YTS	Group2
AS661732	changshamai	hubei	YTS	Group2

Table S1. Details information of 272 natural population materials for genome-wide association analysis.

Accession-ID	Accession-name	Province	Chinese wheat plant zone[a]	Group[b]
AS661739	zhuganqing	hubei	YTS	Group2
AS661746	xiaoganmai	hubei	YTS	Group2
AS661751	tiezimai	hubei	YTS	Group2
AS661755	quanmangxiaomai	hubei	YTS	Group2
AS661756	xishuibagutao	hubei	YTS	Group2
AS661757	zimai	hubei	YTS	Group2
AS661759	wugongmai	hubei	YTS	Group2
AS661762	baikeheshangmai	hunan	YTS	Group2
AS661806	shanyangxue	shaanxi	NW	Group2
AS661814	bailanmai	shaanxi	Y&H	Group2
AS661850	cantiaomai	shaanxi	NW	Group2
AS662012	xiaohongmang	jiangsu	Y&H	Group2
AS662043	bomiduanqu	tibet	Q&T	Group2
WH094	huangshuibai	zhejiang	YTS	Group2
AS661184	cantiaomai	henan	Y&H	Group3
AS661227	liangganbai	henan	YTS	Group3
AS661250	siqiangxiaomai	shaanxi	NW	Group3
AS661254	daqimai	shaanxi	Y&H	Group3
AS661258	yu'ermai	shaanxi	Y&H	Group3
AS661564	puningxiaomai	guangdong	SAS	Group3
AS661571	sanyuehuang	sichuan	SWAS	Group3
AS661582	wuyangmai	sichuan	SWAS	Group3
AS661583	huangmaizi	sichuan	SWAS	Group3
AS661584	yuzuiweimai	sichuan	SWAS	Group3
AS661586	youtiaomai	sichuan	SWAS	Group3
AS661587	dahonghua	sichuan	SWAS	Group3
AS661593	zaohuangmai	sichuan	SWAS	Group3
AS661596	hongtiaomai	sichuan	SWAS	Group3
AS661605	lushanmai	sichuan	SWAS	Group3
AS661609	tuotuomai	sichuan	SWAS	Group3
AS661615	yuweimai	sichuan	SWAS	Group3
AS661616	hechuanmai	sichuan	SWAS	Group3
AS661617	huayangxiaomai	sichuan	SWAS	Group3
AS661619	huanghuaxiaomai	sichuan	SWAS	Group3
AS661620	guangguangtou	sichuan	SWAS	Group3
AS661634	hongmangmaizi	sichuan	Q&T	Group3
AS661637	zaodongmai	sichuan	SWAS	Group3
AS661642	yicuomao	sichuan	SWAS	Group3
AS661658	aikexuxusanyuehuar	sichuan	SWAS	Group3
AS661679	tiekemai	yunnan	SWAS	Group3
AS661683	zimai	yunnan	SWAS	Group3
AS661747	caoxieban	hubei	YTS	Group3
AS661795	huakemai	yunnan	SWAS	Group3
AS661823	baiwugongcao	shaanxi	Y&H	Group3
AS661853	pushanba	shaanxi	YTS	Group3
AS661867	xiaohongmai(babao)	qinghai	Q&T	Group3
AS662061	youmangyangmai	gansu	SWAS	Group3
CS	Chinese Spring	sichuan	SWAS	Group3
AS661094	dabaimai	shanxi	NW	Group4
AS661295	mangmai	inner mongolia	NS	Group4
AS661306	xiaobaisui	inner mongolia	NS	Group4

Table S1. Details information of 272 natural population materials for genome-wide association analysis.

Accession-ID	Accession-name	Province	Chinese wheat plant zone[a]	Group[b]
AS661309	baichunmai	gansu	NWS	Group4
AS661311	laohongmai	gansu	NWS	Group4
AS661320	xiaomai	gansu	NWS	Group4
AS661327	baidatou	gansu	NWS	Group4
AS661329	shanxihong	gansu	NWS	Group4
AS661768	baiyuxiaomai	sichuan	Q&T	Group4
AS661775	aoshanzaoshuxiaom	sichuan	Q&T	Group4
AS661777	zhaonixiaomai	sichuan	SWAS	Group4
AS661778	ranriwumangmai	sichuan	Q&T	Group4
AS661779	qianqianmai	guizhou	SWAS	Group4
AS661791	guangtoubaikemai	yunnan	SWAS	Group4
AS661861	hongmaomai	gansu	NWS	Group4
AS661872	galaohan	qinghai	Q&T	Group4
AS661873	lanmai	qinghai	Q&T	Group4
AS661895	wangkamai	tibet	Q&T	Group4
AS661897	wumangbaimai	tibet	Q&T	Group4
AS661900	zharenmabu	tibet	Q&T	Group4
AS661901	zharenzhuoma	tibet	Q&T	Group4
AS661903	zhaguogouqumai	tibet	Q&T	Group4
AS661904	zhana	tibet	Q&T	Group4
AS661910	maoyinmai	tibet	Q&T	Group4
AS661911	hangmangmaoyinma	tibet	Q&T	Group4
AS661914	changmangpeizhuo	tibet	Q&T	Group4
AS661915	renbuchun	tibet	Q&T	Group4
AS661916	endachangguangma	tibet	Q&T	Group4
AS661934	jiachashumai	tibet	Q&T	Group4
AS661936	jidingwumangchun	tibet	Q&T	Group4
AS661961	suzhuozhuoma	tibet	Q&T	Group4
AS661978	zedangzaxiaomai	tibet	Q&T	Group4
AS662003	wumangxiaomai	tibet	Q&T	Group4
AS662022	shenggexiaomai	tibet	Q&T	Group4
AS662028	zhaxigangzhuo	tibet	Q&T	Group4
AS662033	jigupizhuo	tibet	Q&T	Group4
AS662034	dadangzhuo	tibet	Q&T	Group4
AS662042	yigongzhuo	tibet	Q&T	Group4
AS662049	tajizhuo	tibet	Q&T	Group4
AS662050	pumanghongmai	tibet	Q&T	Group4
AS662052	tarongzhuo	tibet	Q&T	Group4
AS662053	fanshenzhuo	tibet	Q&T	Group4
AS662059	laobaimai	gansu	NWS	Group4
AS662068	hongtuzi	ningxia	NWS	Group4
AS661028	baimai39F	hebei	NW	Mix
AS661067	xiaohongmang	hebei	NW	Mix
AS661117	louguding	shangdong	Y&H	Mix
AS661118	qimai	shangdong	Y&H	Mix
AS661152	yejiling	shangdong	Y&H	Mix
AS661160	shidaosankemang	shangdong	Y&H	Mix
AS661183	dakoumai	henan	Y&H	Mix
AS661209	lingbao925	henan	Y&H	Mix
AS661241	changmangbaike	shaanxi	NW	Mix
AS661260	yangmai	shaanxi	NW	Mix

Table S1. Details information of 272 natural population materials for genome-wide association analysis.

Accession-ID	Accession-name	Province	Chinese wheat plant zone[a]	Group[b]
AS661369	qingxinlanmai	xinjiang	XJ	Mix
AS661599	yupi	sichuan	SWAS	Mix
AS661665	yuqiumai	guizhou	SWAS	Mix
AS661667	caoxiepian	guizhou	SWAS	Mix
AS661668	huimai	guizhou	SWAS	Mix
AS661694	erdaomei	inner mongolia	NS	Mix
AS661724	baisuizi	shangdong	Y&H	Mix
AS661735	baimai	hubei	YTS	Mix
AS661749	yangmai	hubei	YTS	Mix
AS661760	wugongxu	hubei	YTS	Mix
AS661804	xiaohongmai	shaanxi	NW	Mix
AS661855	deguolan	shaanxi	Y&H	Mix
AS661868	xiaohongmai	qinghai	Q&T	Mix
AS661929	jiachazharenzhuoma	tibet	Q&T	Mix
AS661945	quxiajizhuo	tibet	Q&T	Mix
AS661976	bomizamai-5	tibet	Q&T	Mix
AS662020	molengmai	yunnan	SWAS	Mix

Note: [a]. Geographic distribution of the tested landrace accessions. The ten Chinese agro-ecological zones (CAEZs) includes: NW (Northern Winter Wheat Zone), Y&H (Yellow and Huai River Valleys Facultative Wheat Zone), YTS (Middle and Low Yangtze Valleys Autumn-Sown Spring Wheat Zone), SAS (Southern Autumn-Sown Spring Wheat Zone), SWAS (Southwestern Autumn-Sown Spring Wheat Zone), NES (Northeastern Spring Wheat Zone), NS (Northern Spring Wheat Zone), NWS (Northwestern Spring Wheat Zone), Q&T (Qinghai-Tibetan Plateau Spring-Winter Wheat Zone), and XJ (Xinjiang Winter-Spring Wheat Zone). [b]. Groups were culculated by the software Structure 2.3.4
