# Supplementary material for: Multi-Locus Genome-Wide Association Study of Four Yield-Related Traits in Chinese Wheat Landraces
Source: Front Plant Sci. 2021 Aug 16;12:665122. doi: 10.3389/fpls.2021.665122 (PMC8415402; doi:10.3389/fpls.2021.665122)
Supplement: Supplementary file 2 [file Table_2.pdf]

Table S2. Primers sequences of kompetitive allele specific polymerase chain reaction (KASP) markers for *QTkw.sicau-4B* .

QTL	*QTkw.sicau-4B*
Marker	KASP-AX-108886949
Forward primer 1(5'to3')	AGATGTGCCGTGAAATATTGCA
Forward primer 2(5'to3')	AGATGTGCCGTGAAATATTGCG
Reverse primer (5'to3')	CAGCCACCATATACCTGCGA
FAM	GAAGGTGACCAAGTTCATGCT
HEX	GAAGGTCGGAGTCAACGGATT

Note: The probe sequence is not included in the above primer sequence. FAM: probe sequence of the forward primer, and HEX: probe sequence of the reverse primer .
