# Supplementary material for: Multi-Locus Genome-Wide Association Study of Four Yield-Related Traits in Chinese Wheat Landraces
Source: Front Plant Sci. 2021 Aug 16;12:665122. doi: 10.3389/fpls.2021.665122 (PMC8415402; doi:10.3389/fpls.2021.665122)
Supplement: Supplementary file 3 [file Table_3.pdf]

Table S3. Details of significant loci for four yield-related traits detected by five multi-locus GWAS.

| Trait | Marker | Method | Chromosome | Physical Position (Mb) | LOD score | PVE (%) |
|---|---|---|---|---|---|---|
| SL | AX-94847170 | ISIS EM-BLASSO | 1A | 29.61 | 4.13 | 0.99 |
| SL | AX-94847170 | mrMLM | 1A | 29.61 | 5.12 | 2.02 |
| SL | AX-111173409 | pKWmEB | 1A | 458.23 | 3.72 | 1.58 |
| SL | AX-110620420 | ISIS EM-BLASSO | 1A | 566.11 | 7.57 | 3.01 |
| SL | AX-110620420 | mrMLM | 1A | 566.11 | 7.54 | 4.38 |
| SL | AX-110375230 | mrMLM | 1A | 568.36 | 5.63 | 3.14 |
| SL | AX-109320713 | FASTmrEMMA | 1D | 24.06 | 3.45 | 2.34 |
| SL | AX-109320713 | ISIS EM-BLASSO | 1D | 24.06 | 6.86 | 3.29 |
| SL | AX-109320713 | pLARmEB | 1D | 24.06 | 4.65 | 1.36 |
| SL | AX-109886304 | pLARmEB | 2A | 24.11 | 6.15 | 1.46 |
| SL | AX-108833147 | mrMLM | 2A | 197.22 | 5.13 | 5.73 |
| SL | AX-108948074 | ISIS EM-BLASSO | 2A | 735.26 | 10.11 | 5.89 |
| SL | AX-108948074 | pKWmEB | 2A | 735.26 | 11.56 | 7.43 |
| SL | AX-108948074 | pLARmEB | 2A | 735.26 | 5.23 | 1.95 |
| SL | AX-110088953 | ISIS EM-BLASSO | 2A | 742.14 | 4.74 | 4.15 |
| SL | AX-110088953 | mrMLM | 2A | 742.14 | 12.51 | 11.44 |
| SL | AX-111519386 | mrMLM | 2B | 29.19 | 12.03 | 7.77 |
| SL | AX-111519386 | pKWmEB | 2B | 29.19 | 3.47 | 2.24 |
| SL | AX-94545725 | ISIS EM-BLASSO | 2B | 29.71 | 8.45 | 3.97 |
| SL | AX-109906884 | pKWmEB | 2B | 249.67 | 5.00 | 4.73 |
| SL | AX-111083280 | pLARmEB | 2B | 370.43 | 4.81 | 1.98 |
| SL | AX-109424194 | mrMLM | 2B | 534.38 | 7.54 | 9.85 |
| SL | AX-111040830 | pLARmEB | 2B | 643.69 | 9.42 | 2.16 |
| SL | AX-110370760 | ISIS EM-BLASSO | 2B | 647.31 | 4.91 | 2.22 |
| SL | AX-110173655 | FASTmrEMMA | 2B | 733.73 | 7.92 | 5.66 |
| SL | AX-110744761 | pKWmEB | 2D | 36.32 | 5.44 | 1.62 |
| SL | AX-109404693 | pKWmEB | 3A | 23.45 | 3.81 | 2.92 |
| SL | AX-89339669 | FASTmrEMMA | 3A | 650.43 | 6.99 | 3.65 |
| SL | AX-109461933 | ISIS EM-BLASSO | 3A | 650.86 | 14.68 | 4.88 |
| SL | AX-109461933 | mrMLM | 3A | 650.86 | 7.03 | 2.72 |
| SL | AX-109461933 | pKWmEB | 3A | 650.86 | 5.66 | 2.80 |
| SL | AX-110469735 | pLARmEB | 3A | 651.84 | 7.36 | 3.11 |
| SL | AX-95659726 | pKWmEB | 3B | 3.24 | 5.04 | 8.12 |
| SL | AX-109863466 | pLARmEB | 3B | 37.64 | 5.42 | 1.29 |
| SL | AX-108771777 | mrMLM | 3B | 38.80 | 6.20 | 3.65 |
| SL | AX-108771936 | FASTmrEMMA | 3B | 140.13 | 6.51 | 3.19 |
| SL | AX-110372704 | pKWmEB | 3B | 606.57 | 3.65 | 2.80 |
| SL | AX-109536916 | ISIS EM-BLASSO | 3B | 688.40 | 4.00 | 3.16 |
| SL | AX-111953443 | mrMLM | 3D | 592.00 | 3.97 | 2.30 |
| SL | AX-111718558 | FASTmrEMMA | 4A | 47.39 | 5.84 | 3.85 |
| SL | AX-111718558 | pKWmEB | 4A | 47.39 | 6.13 | 4.20 |
| SL | AX-111098528 | mrMLM | 4A | 94.83 | 3.50 | 2.75 |
| SL | AX-109827217 | ISIS EM-BLASSO | 4A | 96.05 | 5.64 | 2.19 |
| SL | AX-109827217 | pKWmEB | 4A | 96.05 | 6.11 | 4.03 |
| SL | AX-109846012 | pKWmEB | 4A | 598.63 | 5.52 | 2.14 |
| SL | AX-108774258 | ISIS EM-BLASSO | 4A | 650.76 | 8.53 | 4.57 |
| SL | AX-108774258 | pLARmEB | 4A | 650.76 | 8.08 | 2.86 |
| SL | AX-109499679 | mrMLM | 4B | 6.94 | 3.77 | 2.48 |
| SL | AX-111107034 | pKWmEB | 4B | 392.37 | 4.78 | 2.65 |
| SL | AX-94673887 | mrMLM | 4D | 222.19 | 3.92 | 2.70 |
| SL | AX-110044071 | pKWmEB | 5A | 596.01 | 4.34 | 1.08 |
| SL | AX-111098595 | mrMLM | 5A | 596.49 | 4.54 | 2.64 |

Table S3. Details of significant loci for four yield-related traits detected by five multi-locus GWAS.

| Trait | Marker | Method | Chromosome | Physical Position (Mb) | LOD score | PVE (%) |
|---|---|---|---|---|---|---|
| SL | AX-109374128 | FASTmrEMMA | 5A | 600.17 | 5.37 | 3.19 |
| SL | AX-109374128 | ISIS EM-BLASSO | 5A | 600.17 | 5.53 | 1.64 |
| SL | AX-110982201 | pKWmEB | 5A | 622.61 | 6.57 | 4.91 |
| SL | AX-109566329 | mrMLM | 5A | 679.75 | 11.34 | 5.75 |
| SL | AX-110012602 | pLARmEB | 5B | 21.61 | 3.31 | 0.29 |
| SL | AX-111658551 | pKWmEB | 5B | 387.35 | 3.75 | 2.46 |
| SL | AX-110395581 | pLARmEB | 5B | 556.06 | 3.75 | 1.49 |
| SL | AX-108919044 | pKWmEB | 5B | 610.29 | 5.69 | 3.08 |
| SL | AX-108776019 | ISIS EM-BLASSO | 5B | 616.97 | 4.29 | 2.40 |
| SL | AX-111627558 | ISIS EM-BLASSO | 5B | 696.99 | 5.60 | 1.82 |
| SL | AX-111627558 | pKWmEB | 5B | 696.99 | 6.25 | 3.33 |
| SL | AX-111616614 | FASTmrEMMA | 5D | 393.80 | 4.23 | 2.26 |
| SL | AX-94781883 | pKWmEB | 6A | 17.64 | 3.49 | 1.67 |
| SL | AX-111484692 | ISIS EM-BLASSO | 6B | 130.34 | 3.92 | 0.94 |
| SL | AX-111288205 | FASTmrEMMA | 6B | 204.48 | 7.82 | 4.15 |
| SL | AX-111288205 | mrMLM | 6B | 204.48 | 4.43 | 2.35 |
| SL | AX-111179390 | mrMLM | 6B | 613.61 | 5.28 | 6.08 |
| SL | AX-109929885 | ISIS EM-BLASSO | 6B | 622.02 | 3.97 | 3.18 |
| SL | AX-111074439 | ISIS EM-BLASSO | 6B | 696.20 | 5.19 | 1.50 |
| SL | AX-109735372 | FASTmrEMMA | 6D | 6.25 | 3.67 | 1.68 |
| SL | AX-111574093 | pKWmEB | 7A | 115.26 | 5.78 | 3.77 |
| SL | AX-109431727 | ISIS EM-BLASSO | 7A | 679.75 | 3.28 | 0.92 |
| SL | AX-109835176 | ISIS EM-BLASSO | 7A | 734.62 | 5.02 | 1.71 |
| SL | AX-86177466 | pKWmEB | 7B | 561.08 | 4.78 | 3.94 |
| SL | AX-109105934 | pLARmEB | 7B | 614.02 | 3.69 | 1.52 |
| SL | AX-110109439 | pKWmEB | 7B | 725.60 | 4.78 | 5.01 |
| SL | AX-109306414 | FASTmrEMMA | 7B | 742.41 | 5.19 | 2.74 |
| SL | AX-109306414 | ISIS EM-BLASSO | 7B | 742.41 | 4.10 | 1.56 |
| SL | AX-109306414 | mrMLM | 7B | 742.41 | 5.34 | 3.11 |
| SL | AX-109306414 | pKWmEB | 7B | 742.41 | 4.04 | 3.05 |
| SL | AX-109881547 | pLARmEB | 7D | 550.55 | 5.17 | 0.72 |
| SN | AX-111487744 | FASTmrEMMA | 1A | 319.89 | 3.47 | 4.43 |
| SN | AX-109460380 | mrMLM | 1A | 378.61 | 5.17 | 5.26 |
| SN | AX-109387367 | pLARmEB | 1A | 380.58 | 5.36 | 8.13 |
| SN | AX-110531273 | ISIS EM-BLASSO | 1A | 584.07 | 3.68 | 1.90 |
| SN | AX-108821334 | pLARmEB | 1B | 313.45 | 4.94 | 3.76 |
| SN | AX-109007688 | pLARmEB | 1B | 613.42 | 8.71 | 4.65 |
| SN | AX-109305103 | ISIS EM-BLASSO | 1B | 630.28 | 8.79 | 2.97 |
| SN | AX-109305103 | mrMLM | 1B | 630.28 | 7.21 | 2.25 |
| SN | AX-109305103 | pKWmEB | 1B | 630.28 | 9.50 | 3.04 |
| SN | AX-109305103 | pLARmEB | 1B | 630.28 | 5.83 | 3.17 |
| SN | AX-108924276 | mrMLM | 2A | 31.70 | 4.30 | 3.60 |
| SN | AX-108924276 | pKWmEB | 2A | 31.70 | 9.41 | 4.33 |
| SN | AX-108831532 | pLARmEB | 2A | 32.54 | 3.70 | 1.45 |
| SN | AX-111594388 | mrMLM | 2A | 524.09 | 8.84 | 7.02 |
| SN | AX-111594388 | pLARmEB | 2A | 524.09 | 4.45 | 5.03 |
| SN | AX-111076227 | ISIS EM-BLASSO | 2A | 524.34 | 7.14 | 4.76 |
| SN | AX-111055038 | FASTmrEMMA | 2A | 606.53 | 4.74 | 3.64 |
| SN | AX-111055038 | ISIS EM-BLASSO | 2A | 606.53 | 4.13 | 1.97 |
| SN | AX-111123457 | ISIS EM-BLASSO | 2A | 741.83 | 5.34 | 3.09 |
| SN | AX-111123457 | pLARmEB | 2A | 741.83 | 4.62 | 4.59 |
| SN | AX-111450513 | pKWmEB | 2A | 741.98 | 8.84 | 5.27 |

Table S3. Details of significant loci for four yield-related traits detected by five multi-locus GWAS.

| Trait | Marker | Method | Chromosome | Physical Position (Mb) | LOD score | PVE (%) |
|---|---|---|---|---|---|---|
| SN | AX-111219617 | mrMLM | 2B | 12.29 | 4.29 | 2.42 |
| SN | AX-89728281 | pLARmEB | 2B | 46.59 | 6.84 | 4.30 |
| SN | AX-108906723 | pKWmEB | 2B | 46.88 | 11.91 | 6.06 |
| SN | AX-111478501 | pKWmEB | 2B | 67.88 | 5.18 | 2.95 |
| SN | AX-109905931 | ISIS EM-BLASSO | 2B | 529.05 | 5.30 | 2.32 |
| SN | AX-109905931 | pKWmEB | 2B | 529.05 | 6.59 | 3.45 |
| SN | AX-111054119 | mrMLM | 2B | 529.29 | 3.65 | 1.11 |
| SN | AX-110604055 | ISIS EM-BLASSO | 2B | 650.16 | 3.51 | 1.66 |
| SN | AX-110604055 | pLARmEB | 2B | 650.16 | 5.33 | 2.85 |
| SN | AX-109340301 | pKWmEB | 2B | 653.01 | 5.14 | 2.40 |
| SN | AX-110360420 | mrMLM | 2D | 297.17 | 5.23 | 4.49 |
| SN | AX-109322933 | pKWmEB | 2D | 601.21 | 3.40 | 1.70 |
| SN | AX-94998712 | mrMLM | 3A | 685.12 | 10.05 | 4.28 |
| SN | AX-89751738 | ISIS EM-BLASSO | 3B | 42.56 | 3.59 | 1.02 |
| SN | AX-109366686 | FASTmrEMMA | 3B | 142.88 | 3.64 | 4.77 |
| SN | AX-109366686 | mrMLM | 3B | 142.88 | 6.51 | 4.05 |
| SN | AX-109582411 | pLARmEB | 3B | 499.75 | 3.85 | 2.37 |
| SN | AX-111017540 | FASTmrEMMA | 3B | 506.98 | 4.00 | 3.45 |
| SN | AX-111017540 | ISIS EM-BLASSO | 3B | 506.98 | 5.33 | 2.33 |
| SN | AX-111606696 | FASTmrEMMA | 3B | 525.23 | 3.57 | 3.46 |
| SN | AX-111606696 | mrMLM | 3B | 525.23 | 6.02 | 5.16 |
| SN | AX-89491115 | mrMLM | 3B | 564.77 | 4.56 | 1.26 |
| SN | AX-108940748 | ISIS EM-BLASSO | 3B | 644.91 | 10.56 | 5.17 |
| SN | AX-108940748 | mrMLM | 3B | 644.91 | 5.60 | 2.96 |
| SN | AX-108940748 | pKWmEB | 3B | 644.91 | 6.95 | 2.90 |
| SN | AX-110422597 | ISIS EM-BLASSO | 3B | 697.13 | 5.28 | 1.32 |
| SN | AX-110439439 | pKWmEB | 3B | 775.34 | 5.02 | 1.53 |
| SN | AX-108757040 | pKWmEB | 3D | 607.09 | 5.95 | 4.00 |
| SN | AX-111723748 | mrMLM | 4A | 52.61 | 10.04 | 13.09 |
| SN | AX-111707709 | mrMLM | 4A | 92.21 | 3.52 | 4.19 |
| SN | AX-108767449 | pKWmEB | 4A | 626.02 | 8.25 | 5.29 |
| SN | AX-110994064 | pLARmEB | 4B | 13.43 | 3.13 | 1.61 |
| SN | AX-94727532 | pLARmEB | 4D | 395.38 | 6.96 | 6.57 |
| SN | AX-110188531 | pKWmEB | 5A | 217.90 | 4.53 | 3.85 |
| SN | AX-109976071 | pKWmEB | 5A | 565.49 | 4.40 | 3.41 |
| SN | AX-110991372 | mrMLM | 5A | 589.22 | 5.85 | 6.33 |
| SN | AX-108842302 | pKWmEB | 5A | 678.62 | 4.31 | 1.39 |
| SN | AX-109538487 | ISIS EM-BLASSO | 5A | 678.94 | 4.55 | 1.64 |
| SN | AX-110560000 | mrMLM | 5A | 680.92 | 6.26 | 4.49 |
| SN | AX-110560000 | pKWmEB | 5A | 680.92 | 4.36 | 2.83 |
| SN | AX-109409751 | FASTmrEMMA | 5A | 682.90 | 3.07 | 3.43 |
| SN | AX-109816265 | mrMLM | 5A | 682.92 | 5.63 | 2.83 |
| SN | AX-109348429 | ISIS EM-BLASSO | 5B | 404.48 | 4.90 | 3.91 |
| SN | AX-109348429 | pKWmEB | 5B | 404.48 | 7.73 | 8.09 |
| SN | AX-109513893 | ISIS EM-BLASSO | 5B | 487.29 | 6.08 | 1.62 |
| SN | AX-108914525 | ISIS EM-BLASSO | 6A | 445.42 | 4.92 | 2.78 |
| SN | AX-108914525 | pLARmEB | 6A | 445.42 | 4.92 | 3.60 |
| SN | AX-109469885 | pKWmEB | 6A | 469.11 | 3.34 | 0.86 |
| SN | AX-109370998 | pKWmEB | 6B | 55.34 | 4.55 | 2.12 |
| SN | AX-111494120 | FASTmrEMMA | 6B | 114.08 | 6.97 | 4.73 |
| SN | AX-110413210 | ISIS EM-BLASSO | 6B | 116.60 | 7.16 | 3.90 |
| SN | AX-110027598 | ISIS EM-BLASSO | 6B | 397.51 | 5.31 | 1.59 |

Table S3. Details of significant loci for four yield-related traits detected by five multi-locus GWAS.

| Trait | Marker | Method | Chromosome | Physical Position (Mb) | LOD score | PVE (%) |
|---|---|---|---|---|---|---|
| SN | AX-108781775 | ISIS EM-BLASSO | 6B | 658.65 | 6.53 | 2.56 |
| SN | AX-108781775 | pKWmEB | 6B | 658.65 | 3.94 | 2.43 |
| SN | AX-110097737 | pLARmEB | 6D | 54.08 | 5.59 | 1.88 |
| SN | AX-111405245 | ISIS EM-BLASSO | 6D | 68.56 | 4.05 | 1.23 |
| SN | AX-110708019 | mrMLM | 7A | 670.88 | 5.31 | 1.98 |
| SN | AX-110931532 | ISIS EM-BLASSO | 7A | 671.48 | 7.39 | 5.13 |
| SN | AX-110931532 | pKWmEB | 7A | 671.48 | 15.85 | 7.61 |
| SN | AX-111600553 | ISIS EM-BLASSO | 7A | 671.48 | 4.07 | 2.83 |
| SN | AX-111600553 | pLARmEB | 7A | 671.48 | 4.81 | 5.44 |
| SN | AX-110956517 | pKWmEB | 7A | 680.27 | 6.29 | 2.87 |
| SN | AX-109425504 | ISIS EM-BLASSO | 7A | 733.84 | 13.18 | 4.79 |
| SN | AX-111092457 | mrMLM | 7A | 734.07 | 4.06 | 1.94 |
| SN | AX-109825419 | pKWmEB | 7B | 79.29 | 6.81 | 3.34 |
| SN | AX-109869759 | mrMLM | 7B | 587.91 | 6.42 | 4.76 |
| SN | AX-111475579 | ISIS EM-BLASSO | 7B | 664.10 | 3.99 | 1.00 |
| SN | AX-109435062 | pKWmEB | 7B | 702.45 | 4.37 | 1.97 |
| SN | AX-111211668 | FASTmrEMMA | 7D | 605.26 | 5.13 | 4.75 |
| SN | AX-111211668 | pKWmEB | 7D | 605.26 | 7.01 | 2.08 |
| TKW | AX-111638647 | ISIS EM-BLASSO | 1A | 10.47 | 3.34 | 1.85 |
| TKW | AX-108830326 | mrMLM | 1A | 514.68 | 3.03 | 3.65 |
| TKW | AX-109038078 | pKWmEB | 1B | 604.66 | 3.29 | 4.34 |
| TKW | AX-95124839 | pLARmEB | 1D | 28.17 | 6.69 | 6.16 |
| TKW | AX-110393778 | mrMLM | 1D | 64.77 | 5.53 | 2.90 |
| TKW | AX-110823145 | ISIS EM-BLASSO | 1D | 424.70 | 4.31 | 1.67 |
| TKW | AX-108811726 | FASTmrEMMA | 2A | 5.70 | 4.65 | 2.21 |
| TKW | AX-110983850 | FASTmrEMMA | 2A | 765.86 | 3.60 | 2.85 |
| TKW | AX-111113548 | ISIS EM-BLASSO | 2B | 50.73 | 9.88 | 6.90 |
| TKW | AX-111113548 | pKWmEB | 2B | 50.73 | 5.39 | 5.97 |
| TKW | AX-111213423 | FASTmrEMMA | 2B | 50.77 | 3.37 | 1.91 |
| TKW | AX-111168305 | ISIS EM-BLASSO | 2B | 566.40 | 6.86 | 3.56 |
| TKW | AX-111168305 | pKWmEB | 2B | 566.40 | 3.12 | 2.57 |
| TKW | AX-108800291 | FASTmrEMMA | 2B | 600.70 | 6.66 | 4.94 |
| TKW | AX-110129867 | pKWmEB | 2B | 651.61 | 3.76 | 3.35 |
| TKW | AX-108909788 | pLARmEB | 2B | 651.73 | 3.63 | 1.96 |
| TKW | AX-109558762 | ISIS EM-BLASSO | 2D | 22.16 | 4.62 | 1.34 |
| TKW | AX-95003297 | FASTmrEMMA | 3A | 686.12 | 4.16 | 2.13 |
| TKW | AX-95003297 | ISIS EM-BLASSO | 3A | 686.12 | 5.35 | 2.35 |
| TKW | AX-95003297 | mrMLM | 3A | 686.12 | 5.39 | 3.02 |
| TKW | AX-95003297 | pKWmEB | 3A | 686.12 | 9.22 | 4.65 |
| TKW | AX-95003297 | pLARmEB | 3A | 686.12 | 8.72 | 3.69 |
| TKW | AX-86170856 | pKWmEB | 3A | 725.74 | 3.58 | 4.95 |
| TKW | AX-109466851 | pKWmEB | 3B | 498.06 | 5.65 | 2.40 |
| TKW | AX-111730285 | FASTmrEMMA | 3B | 580.03 | 5.23 | 2.51 |
| TKW | AX-111179542 | mrMLM | 3B | 773.90 | 7.37 | 8.91 |
| TKW | AX-109328192 | pLARmEB | 4A | 541.43 | 4.77 | 2.33 |
| TKW | AX-108726977 | mrMLM | 4A | 616.61 | 7.22 | 5.45 |
| TKW | AX-108726977 | pLARmEB | 4A | 616.61 | 3.94 | 1.73 |
| TKW | AX-109980998 | mrMLM | 4B | 543.86 | 3.81 | 7.14 |
| TKW | AX-108886949 | ISIS EM-BLASSO | 4B | 553.54 | 8.59 | 9.27 |
| TKW | AX-108886949 | pKWmEB | 4B | 553.54 | 12.04 | 18.78 |
| TKW | AX-108886949 | pLARmEB | 4B | 553.54 | 10.20 | 11.09 |
| TKW | AX-110063521 | mrMLM | 4B | 663.69 | 3.36 | 2.65 |

Table S3. Details of significant loci for four yield-related traits detected by five multi-locus GWAS.

| Trait | Marker | Method | Chromosome | Physical Position (Mb) | LOD score | PVE (%) |
|---|---|---|---|---|---|---|
| TKW | AX-109276303 | FASTmrEMMA | 5B | 412.60 | 5.58 | 2.22 |
| TKW | AX-111059020 | ISIS EM-BLASSO | 5B | 415.16 | 3.42 | 3.33 |
| TKW | AX-108828326 | mrMLM | 5B | 488.71 | 4.23 | 6.26 |
| TKW | AX-109305922 | pKWmEB | 5B | 695.99 | 6.02 | 2.89 |
| TKW | AX-111173397 | pLARmEB | 5D | 385.80 | 4.39 | 0.86 |
| TKW | AX-95003025 | pLARmEB | 5D | 477.83 | 4.34 | 1.69 |
| TKW | AX-111086205 | FASTmrEMMA | 6B | 201.58 | 7.51 | 3.54 |
| TKW | AX-111086205 | ISIS EM-BLASSO | 6B | 201.58 | 3.81 | 1.39 |
| TKW | AX-111086205 | mrMLM | 6B | 201.58 | 4.21 | 2.72 |
| TKW | AX-111086205 | pKWmEB | 6B | 201.58 | 8.80 | 5.12 |
| TKW | AX-111474814 | ISIS EM-BLASSO | 6B | 675.56 | 3.90 | 1.88 |
| TKW | AX-109748235 | mrMLM | 6D | 469.29 | 10.08 | 8.66 |
| TKW | AX-109748235 | pLARmEB | 6D | 469.29 | 5.03 | 2.85 |
| TKW | AX-111464050 | ISIS EM-BLASSO | 7A | 51.41 | 4.05 | 1.88 |
| TKW | AX-111490904 | FASTmrEMMA | 7A | 52.36 | 6.55 | 4.25 |
| TKW | AX-109033661 | ISIS EM-BLASSO | 7A | 89.32 | 5.19 | 3.18 |
| TKW | AX-109033661 | mrMLM | 7A | 89.32 | 9.40 | 6.41 |
| TKW | AX-109033661 | pKWmEB | 7A | 89.32 | 4.58 | 4.72 |
| TKW | AX-109832429 | pKWmEB | 7A | 678.58 | 4.87 | 2.24 |
| TKW | AX-110378610 | ISIS EM-BLASSO | 7A | 696.41 | 5.79 | 5.10 |
| TKW | AX-110378610 | pKWmEB | 7A | 696.41 | 4.72 | 4.71 |
| TKW | AX-110378610 | pLARmEB | 7A | 696.41 | 8.35 | 5.63 |
| TKW | AX-110628451 | mrMLM | 7A | 723.26 | 3.85 | 2.92 |
| TKW | AX-111161343 | pKWmEB | 7A | 736.53 | 4.00 | 5.71 |
| TKW | AX-110516887 | mrMLM | 7B | 4.92 | 5.97 | 4.02 |
| TKW | AX-110982569 | FASTmrEMMA | 7B | 517.43 | 5.30 | 2.75 |
| TKW | AX-110982569 | mrMLM | 7B | 517.43 | 6.25 | 3.03 |
| TKW | AX-110982569 | pLARmEB | 7B | 517.43 | 7.16 | 2.99 |
| TKW | AX-94474770 | ISIS EM-BLASSO | 7B | 701.21 | 3.70 | 2.80 |
| TKW | AX-109997331 | pLARmEB | 7B | 725.84 | 5.68 | 3.40 |
| TKW | AX-110016119 | FASTmrEMMA | 7B | 726.39 | 4.48 | 2.60 |
| TKW | AX-110016119 | ISIS EM-BLASSO | 7B | 726.39 | 4.97 | 2.17 |
| TKW | AX-109353584 | pLARmEB | 7D | 18.41 | 4.19 | 1.34 |
| TKW | AX-94824164 | mrMLM | 7D | 29.88 | 3.47 | 5.23 |
| TKW | AX-110472881 | pLARmEB | 7D | 532.03 | 4.55 | 1.59 |
| TN | AX-109935792 | pKWmEB | 1A | 39.15 | 6.44 | 8.94 |
| TN | AX-110925917 | pLARmEB | 1A | 564.87 | 4.14 | 1.38 |
| TN | AX-109582231 | FASTmrEMMA | 1B | 21.47 | 3.28 | 1.81 |
| TN | AX-109582231 | ISIS EM-BLASSO | 1B | 21.47 | 3.01 | 1.40 |
| TN | AX-109582231 | mrMLM | 1B | 21.47 | 4.47 | 4.38 |
| TN | AX-109582231 | pKWmEB | 1B | 21.47 | 5.12 | 2.93 |
| TN | AX-109582231 | pLARmEB | 1B | 21.47 | 4.60 | 2.12 |
| TN | AX-109872621 | ISIS EM-BLASSO | 1B | 418.16 | 5.80 | 5.25 |
| TN | AX-111045160 | ISIS EM-BLASSO | 1B | 585.32 | 4.92 | 3.45 |
| TN | AX-111045160 | pKWmEB | 1B | 585.32 | 6.55 | 4.75 |
| TN | AX-111954129 | mrMLM | 1D | 47.65 | 5.02 | 3.69 |
| TN | AX-110416914 | mrMLM | 2A | 27.47 | 3.63 | 4.67 |
| TN | AX-109381037 | pKWmEB | 2A | 27.72 | 7.51 | 6.00 |
| TN | AX-111450090 | mrMLM | 2A | 151.26 | 5.52 | 7.10 |
| TN | AX-109440041 | FASTmrEMMA | 2A | 605.30 | 6.15 | 6.40 |
| TN | AX-109440041 | pKWmEB | 2A | 605.30 | 3.72 | 4.18 |
| TN | AX-108888319 | ISIS EM-BLASSO | 2A | 719.31 | 3.79 | 2.63 |

Table S3. Details of significant loci for four yield-related traits detected by five multi-locus GWAS.

| Trait | Marker | Method | Chromosome | Physical Position (Mb) | LOD score | PVE (%) |
|---|---|---|---|---|---|---|
| TN | AX-108888319 | pKWmEB | 2A | 719.31 | 4.36 | 2.57 |
| TN | AX-110497091 | pKWmEB | 2A | 753.70 | 4.98 | 2.10 |
| TN | AX-110093452 | ISIS EM-BLASSO | 2B | 10.99 | 4.27 | 3.04 |
| TN | AX-110093452 | pLARmEB | 2B | 10.99 | 6.10 | 3.87 |
| TN | AX-110407616 | pKWmEB | 2B | 10.99 | 7.76 | 3.88 |
| TN | AX-111717848 | ISIS EM-BLASSO | 2B | 110.49 | 3.42 | 4.20 |
| TN | AX-110025635 | pKWmEB | 2B | 644.85 | 3.11 | 2.64 |
| TN | AX-110424541 | pKWmEB | 2B | 729.65 | 3.53 | 2.13 |
| TN | AX-110471898 | mrMLM | 2D | 20.33 | 6.56 | 5.11 |
| TN | AX-110471898 | pLARmEB | 2D | 20.33 | 3.01 | 0.93 |
| TN | AX-109994590 | pLARmEB | 2D | 502.03 | 3.46 | 1.12 |
| TN | AX-110950815 | mrMLM | 3A | 421.77 | 3.92 | 3.92 |
| TN | AX-110496125 | FASTmrEMMA | 3A | 685.44 | 3.48 | 1.86 |
| TN | AX-111622188 | ISIS EM-BLASSO | 3A | 685.81 | 4.97 | 2.76 |
| TN | AX-111591456 | pKWmEB | 3A | 721.38 | 9.81 | 7.43 |
| TN | AX-108844766 | FASTmrEMMA | 3A | 722.34 | 3.00 | 2.15 |
| TN | AX-110195172 | ISIS EM-BLASSO | 3A | 740.87 | 4.83 | 4.08 |
| TN | AX-108901759 | pKWmEB | 3B | 17.81 | 3.99 | 2.41 |
| TN | AX-109876424 | ISIS EM-BLASSO | 3B | 17.93 | 4.28 | 1.94 |
| TN | AX-111456619 | pKWmEB | 3B | 74.00 | 4.00 | 4.24 |
| TN | AX-109013525 | mrMLM | 3B | 239.92 | 3.39 | 8.42 |
| TN | AX-109013525 | pLARmEB | 3B | 239.92 | 3.11 | 3.00 |
| TN | AX-109981840 | mrMLM | 3B | 667.17 | 4.45 | 4.49 |
| TN | AX-110502357 | pLARmEB | 4B | 653.13 | 5.81 | 3.33 |
| TN | AX-109427123 | ISIS EM-BLASSO | 5B | 605.53 | 5.30 | 2.12 |
| TN | AX-109427123 | mrMLM | 5B | 605.53 | 4.43 | 3.56 |
| TN | AX-109427123 | pKWmEB | 5B | 605.53 | 3.14 | 1.74 |
| TN | AX-111917292 | pLARmEB | 5D | 38.93 | 3.95 | 2.04 |
| TN | AX-108836317 | pKWmEB | 6A | 535.81 | 4.76 | 5.45 |
| TN | AX-109640953 | mrMLM | 6B | 190.57 | 6.89 | 6.58 |
| TN | AX-108789690 | pLARmEB | 6B | 195.12 | 4.48 | 2.44 |
| TN | AX-110963009 | FASTmrEMMA | 6B | 207.45 | 7.54 | 6.55 |
| TN | AX-110963009 | pKWmEB | 6B | 207.45 | 8.37 | 5.39 |
| TN | AX-111739366 | ISIS EM-BLASSO | 6B | 446.83 | 4.44 | 6.06 |
| TN | AX-109829659 | mrMLM | 6B | 647.93 | 3.57 | 3.22 |
| TN | AX-110969084 | FASTmrEMMA | 6D | 469.60 | 3.77 | 3.70 |
| TN | AX-110989608 | ISIS EM-BLASSO | 6D | 472.57 | 4.26 | 2.96 |
| TN | AX-110989608 | pKWmEB | 6D | 472.57 | 8.42 | 6.73 |
| TN | AX-111919829 | mrMLM | 6D | 472.69 | 8.20 | 9.58 |
| TN | AX-109296056 | pLARmEB | 6D | 472.74 | 6.04 | 3.53 |
| TN | AX-108923376 | mrMLM | 7A | 5.43 | 3.67 | 1.69 |
| TN | AX-109036550 | pKWmEB | 7A | 652.49 | 4.69 | 2.08 |
| TN | AX-111651884 | ISIS EM-BLASSO | 7A | 691.26 | 3.33 | 0.86 |
| TN | AX-109597801 | ISIS EM-BLASSO | 7B | 52.12 | 4.87 | 3.44 |
| TN | AX-111485072 | ISIS EM-BLASSO | 7B | 742.13 | 3.97 | 3.02 |
| TN | AX-111374877 | pLARmEB | 7D | 191.82 | 3.53 | 1.12 |
| TN | AX-111576325 | pKWmEB | 7D | 221.25 | 4.15 | 1.83 |
| TN | AX-111498905 | mrMLM | 7D | 263.88 | 3.45 | 2.52 |

Note: SL, spike length; SN, spikelets number per spike; TN, tillers number; TKW, thousand-kernel weight; LOD, logarithm of odds; PVE, phenotypic variation explained.
