# Supplementary figures and images for: Multi-Locus Genome-Wide Association Study of Four Yield-Related Traits in Chinese Wheat Landraces
Source: Front Plant Sci. 2021 Aug 16;12:665122. doi: 10.3389/fpls.2021.665122 (PMC8415402; doi:10.3389/fpls.2021.665122)

**Figure S1.** The high expression level of predict gene expression on *QTKw.sicau-4B*.

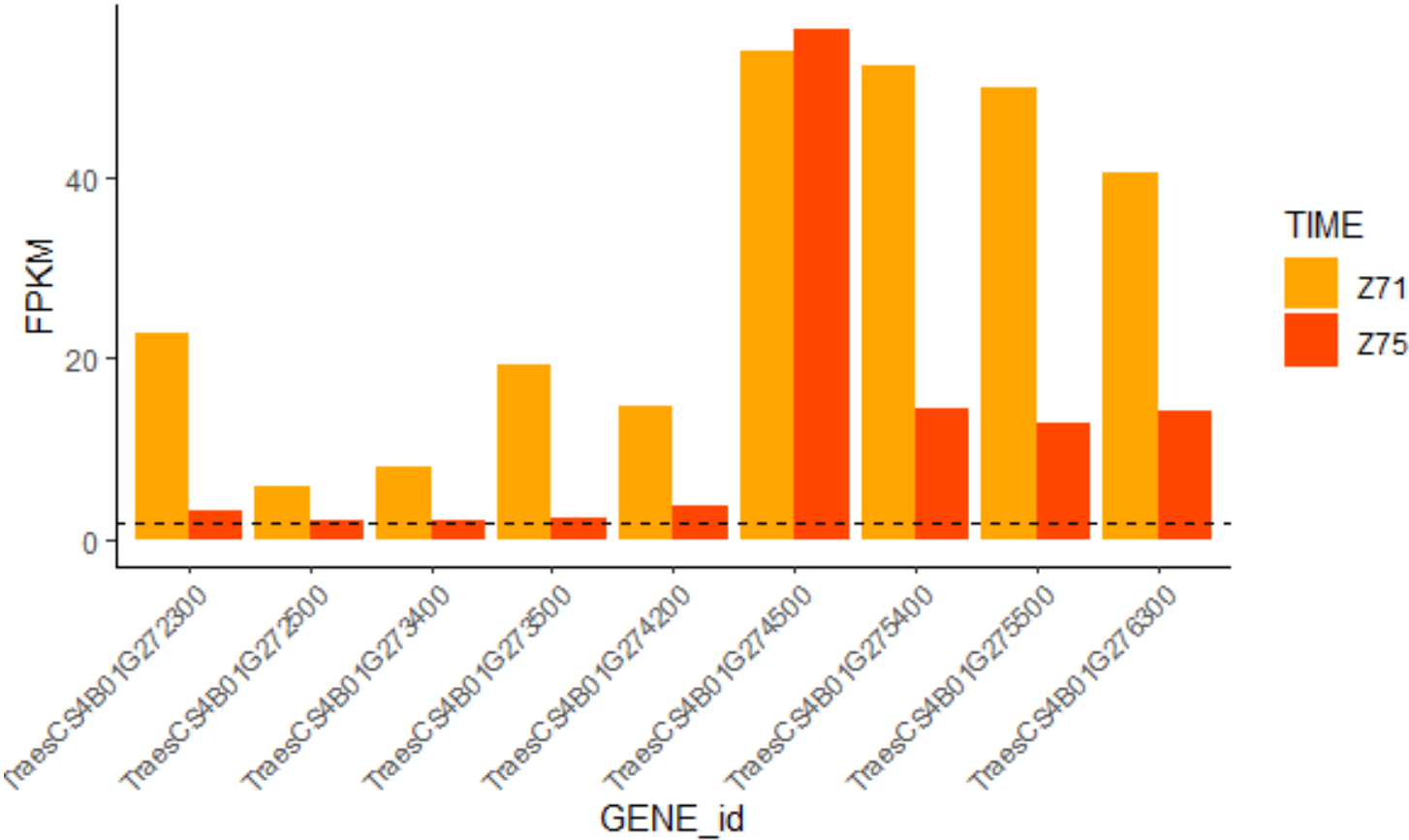

Supplement: Supplementary file 4 [file Image_1.pdf]
